# PolyGlcNAc-containing exopolymers enable surface penetration by non-motile *Enterococcus faecalis*

**DOI:** 10.1371/journal.ppat.1007571

**Published:** 2019-02-11

**Authors:** Yusibeska Ramos, Jorge Rocha, Ana L. Hael, Jordi van Gestel, Hera Vlamakis, Colette Cywes-Bentley, Juan R. Cubillos-Ruiz, Gerald B. Pier, Michael S. Gilmore, Roberto Kolter, Diana K. Morales

**Affiliations:** 1 Department of Obstetrics and Gynecology, Weill Cornell Medicine, New York, NY, United States of America; 2 Department of Microbiology and Immunobiology, Harvard Medical School, Boston, MA, United States of America; 3 Department of Evolutionary Biology and Environmental Studies, University of Zürich, Zürich, Switzerland; 4 Swiss Institute of Bioinformatics, Lausanne, Switzerland; 5 Department of Environmental Microbiology, Swiss Federal Institute of Aquatic Science and Technology (Eawag), Dübendorf, Switzerland; 6 Department of Environmental Systems Science, ETH Zürich, Zürich, Switzerland; 7 Division of Infectious Diseases, Department of Medicine, Brigham and Women’s Hospital, Harvard Medical School, Boston, MA, United States of America; 8 Department of Ophthalmology, Harvard Medical School, Boston, MA, United States of America; University of Kansas, UNITED STATES

## Abstract

Bacterial pathogens have evolved strategies that enable them to invade tissues and spread within the host. *Enterococcus faecalis* is a leading cause of local and disseminated multidrug-resistant hospital infections, but the molecular mechanisms used by this non-motile bacterium to penetrate surfaces and translocate through tissues remain largely unexplored. Here we present experimental evidence indicating that *E*. *faecalis* generates exopolysaccharides containing β-1,6-linked poly-*N*-acetylglucosamine (polyGlcNAc) as a mechanism to successfully penetrate semisolid surfaces and translocate through human epithelial cell monolayers. Genetic screening and molecular analyses of mutant strains identified *glnA*, *rpiA* and *epaX* as genes critically required for optimal *E*. *faecalis* penetration and translocation. Mechanistically, GlnA and RpiA cooperated to generate uridine diphosphate *N*-acetylglucosamine (UDP-GlcNAc) that was utilized by EpaX to synthesize polyGlcNAc-containing polymers. Notably, exogenous supplementation with polymeric *N*-acetylglucosamine (PNAG) restored surface penetration by *E*. *faecalis* mutants devoid of EpaX. Our study uncovers an unexpected mechanism whereby the RpiA-GlnA-EpaX metabolic axis enables production of polyGlcNAc-containing polysaccharides that endow *E*. *faecalis* with the ability to penetrate surfaces. Hence, targeting carbohydrate metabolism or inhibiting biosynthesis of polyGlcNAc-containing exopolymers may represent a new strategy to more effectively confront enterococcal infections in the clinic.

## Author summary

*Enterococcus faecalis* is a microbial inhabitant of the human gastrointestinal tract that can cause lethal infections. Typically classified as a non-motile bacterium, *E*. *faecalis* can readily migrate and translocate across epithelial barriers to invade distant organs. Nevertheless, the molecular pathways driving enterococcal invasive attributes remain poorly understood. In this study, we uncover that *E*. *faecalis* produces a polyGlcNAc-containing extracellular glycopolymer to efficiently migrate into semisolid surfaces and translocate through human epithelial cell monolayers. Our work provides evidence that non-motile bacterial pathogens can exploit endogenous carbohydrate metabolic pathways to penetrate surfaces. Thus, targeting glycopolymer biosynthetic programs might be useful to control infections by Gram-positive cocci in the clinic.

## Introduction

Microbes use a variety of strategies to obtain nutrients and ensure survival. While motility could be used as a means for accessing nutrient sources, non-motile bacterial species require unconventional mechanisms to accomplish this goal. *Enterococcus faecalis* is a non-motile, facultative anaerobic bacterium that inhabits the human gastrointestinal (GI) tract [1]. However, hypervirulent *E*. *faecalis* strains resistant to multiple antibiotics often cause hospital-acquired urinary tract, wound and abdominal infections, as well as bacteremia and infective endocarditis [1]. Enterococci can adhere to and invade host tissues in order to act as lethal pathogens. Indeed, *E*. *faecalis* translocation across the intestinal barrier enables bacterial spread and colonization of distal anatomical sites [2, 3]. Interestingly, *E*. *faecalis* extra-intestinal translocation appears to be promoted by association with epithelial cells in aggregates [3], a process that is partly mediated by the synthesis of adhesins [3–5] and additional unknown factors.

Enterococci produce diverse cell wall-anchored polysaccharides [6–8], which generally consist of repeating units of oligosaccharides that are associated with bacterial surfaces through linkage to cell membrane, peptidoglycan or other unknown mechanisms [7, 9]. *E*. *faecalis* displays an extensive surface glycome, including wall teichoic acid and lipoteichoic acid polymers, capsular polysaccharides [7, 10], and the enterococcal polysaccharide antigen (EPA), which is a rhamnopolysaccharide. EPA, mainly composed of glucose, rhamnose, *N*-acetylglucosamine (GlcNAc), *N*-acetylgalactosamine (GalNAc), and galactose that appears to be buried within the cell wall, thus precluding interaction with host cells [7]. In addition to these polysaccharides, a new glycopolymer was recently discovered on the surface of *E*. *faecalis* cells by immunofluorescence assays using the human IgG monoclonal antibody (mAb) F598, which specifically binds to β-1,6-linked GlcNAc polysaccharides [8, 11]. While this putative polyGlcNAc-like polymer has not been studied in *E*. *faecalis*, other reports have characterized similar polysaccharides that react with mAb F598 [12, 13], termed either PIA (for polysaccharide intercellular adhesin) in *Staphylococcus epidermidis* [14, 15] or PNAG (for polymeric *N*-acetyl glucosamine) in *Staphylococcus aureus* and other pathogens [16, 17]. Of note, these extracellular glycopolymers consist of β-1,6-linked GlcNAc residues containing 5–10% positively-charged amino groups (due to partial de-*N*-acetylation (GlcNH_3_)) as well as negative charges (resulting from O-succinylation) [8, 14–17].

*E*. *faecalis* has been shown to invade surfaces such as mammalian tissues [3, 18] and penetrate solid culture media [19], but the mechanisms driving these processes remain elusive. In the present study, we identified the molecular events and metabolic pathways that endow *E*. *faecalis* with remarkable capacity to penetrate semisolid surfaces. We found that *E*. *faecalis* produces extracellular polyGlcNAc-containing polymers to form penetrating microcolonies inside semisolid surfaces. Using diverse genetic and biochemical approaches, we determined that biosynthesis of these complex exopolymers occurs through the RpiA-GlnA-EpaX metabolic pathway. Notably, *E*. *faecalis* mutants unable to produce polyGlcNAc-containing polymers demonstrated impaired capacity to pass into semisolid surfaces and translocate through human epithelial cell monolayers.

## Results

### The non-motile bacterium *E*. *faecalis* penetrates semisolid surfaces

**Fig 1:**
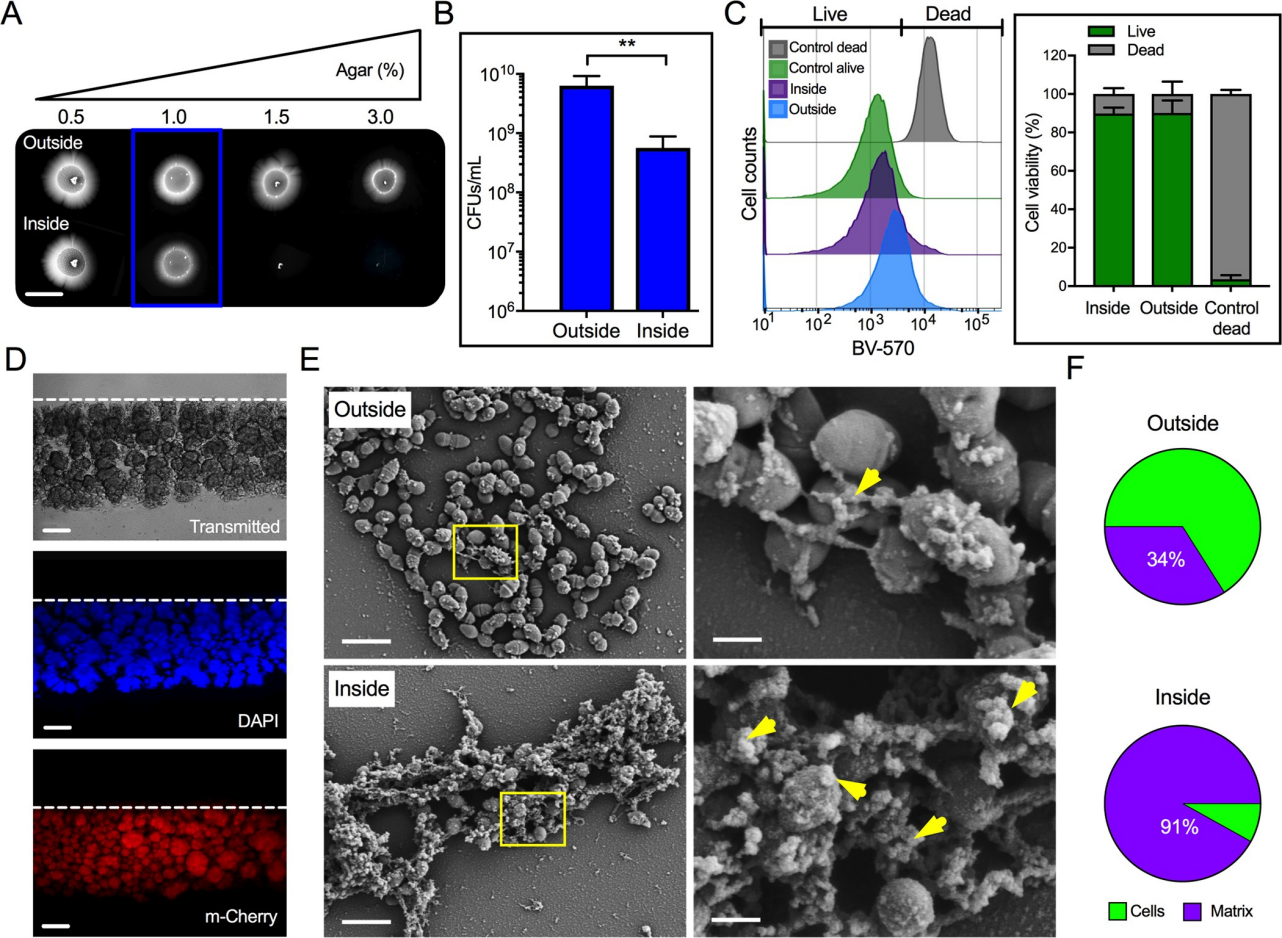
*E*. *faecalis* cells penetrating semisolid media are covered by an extracellular matrix. (**A—F**) The capacity of *E*. *faecalis* MMH594 to penetrate semisolid MOLP was evidenced as a colony-print inside the agar after removing external non-penetrating cells. (**A**) Macroscopic images of colonies (outside) and penetrating bacteria (inside) on solidified medium with the indicated agar concentrations. Scale bar: 6,000 μm. (**B**) Quantification of internal and external cells grown on semisolid MOLP (1% agar; *blue box*) expressed as CFUs/mL (mean±SE; n = 6; **P<0.01; two-tailed unpaired *t*–test with Mann-Whitney test). Data are representative of 5 independent experiments. (**C**) FACS-based viability analysis of penetrating and non-penetrating *E*. *faecalis* (*right*; mean±SE; n = 8). (**D**) Side sections of *E*. *faecalis* inside semisolid MOLP as described above. Top dashed lines indicate the beginning of the agar in each section. Lower scale bar: 60 μm. (**E**) SEM images of aggregated and matrix-covered (*yellow arrow head*) invading and non-invading cells (*right panels*; scale bar: 400 nm). Yellow square areas were imaged at higher magnifications (*left panels*; scale bar: 2 μm). (**F**) Fractions of cells and extracellular matrix found on the surface (~111 μm^2^) visible in the SEM images of penetrating and non-penetrating bacteria, as quantified using automated image analysis (mean±SE; n = 3; *P* < 0.05; two-tailed unpaired *t*–test).

Analysis of semisolid media penetration has been useful for identifying and characterizing virulence traits in human fungal pathogens [20–23]. We sought to exploit this approach to understand the molecular mechanisms that *E*. *faecalis* utilizes to enter surfaces. Under our conditions, an indelible bacterial “colony-print” developed inside modified MOLP (medium optimal for lipopeptide production) [24], when six-day-old colonies of the clinical isolate *E*. *faecalis* were extensively washed with water to remove adventitiously associated bacterial cells from the surface of the agar. This colony-print, indicative of penetration, was not observed at agar concentrations above 1.0% (Fig 1A), and agar degradation was not evidenced once the external (non-penetrating) cells were removed. Importantly, we identified this semisolid agar-penetration trait in several clinical isolates as well as in the commensal-like strain *E*. *faecalis* OG1RF (S1A Fig). Kinetic analyses revealed that *E*. *faecalis* agar entrance was macroscopically evident 48 h post-inoculation, and that it progressed concomitantly with colony expansion (S1B Fig). Of note, the agar penetration ability of all *E*. *faecalis* strains tested in this study was not lost upon laboratory domestication. This phenomenon was not only evidenced when *E*. *faecalis* colonies were grown on culture media solidified with agarose, but also with the copolymer poloxamer-407 (MOLP-407) (S1C and S1D Fig; *top*), demonstrating that this process is not specific to the nature of the gelling agent.

To quantify the number of penetrating and non-penetrating bacteria, colony forming units (CFUs) were determined from both inside and outside *E*. *faecalis* populations grown on semisolid MOLP. Some of the penetrating bacteria were able to form colonies, but the total number of CFUs obtained for this population (~5x10^8^) was reproducibly one order of magnitude lower than that of the external cells (~6x10^9^; Fig 1B). Similarly, the total CFUs obtained from the penetrating population grown on MOLP-407 were lower (~2x10^8^) than the CFUs obtained for external cells (~1x10^9^; S1D Fig, *bottom*). Flow cytometric analyses of penetrating and non-penetrating bacteria grown on MOLP were performed to determine the viability of these cells after staining with the live/dead dye Brilliant Violet-570 (BV-570). In contrast to the heat-killed control showing less than 5% viable cells, ~90% of the penetrating and non-penetrating population were viable under our culture conditions (Fig 1C). Together, these data indicate that *E*. *faecalis* can pass into semisolid surfaces and that the majority of the penetrating cells remain viable during this process.

### The colony-print is formed by bacterial microcolonies covered by an extracellular matrix

To further understand the *E*. *faecalis* penetration process, we analyzed agar side sections of approximately 2 mm-wide obtained from six-day-old colony-prints produced by penetrating bacteria grown in MOLP. Interestingly, isolated aggregates with varying morphologies and sizes readily formed inside the agar (Fig 1D; *top*). Penetration was found to decrease proportionally from the center (S1E Fig; depth of ~128 μm) to the edge (S1F Fig; depth of ~6 μm) of the colony, with several aggregates penetrating deeper at the center (Fig 1D; *top* and S1E Fig). DAPI staining (Fig 1D; *middle*) and epifluorescence analysis of penetrating bacterial cells constitutively expressing m-Cherry (Fig 1D; *bottom*) confirmed that these internal clumps contained viable and metabolically active *E*. *faecalis* cells.

Scanning electron microscopy (SEM) analyses of *E*. *faecalis* colonies were performed to determine the morphological status of external and MOLP-penetrating cells. No major changes in cell morphology were found and only normal diplococcal, clumped or isolated, bacterial cells were observed. Strikingly, however, cells within the aggregates were covered with and connected by an extracellular matrix that appeared to be more abundantly produced by invading cells than surface cells (Fig 1E). Indeed, automated SEM image analysis (see Material and Methods) determined that internal cells exhibited significantly higher matrix coverage than external cells (Fig 1F). These data indicate that *E*. *faecalis* penetrates the agar surface and that this process is accompanied by the generation of microcolonies formed by matrix-covered cells.

### *E*. *faecalis* mutants defective in semisolid surface penetration

Cellular structures, such as pili, have been shown to be involved in mediating Gram-positive bacterial motility [25]. To determine whether pili expression could mediate the penetration process observed, we tested two previously generated pili-deficient *E*. *faecalis* mutants (Δ*ebpABC* and Δ*ebpA*), and their parental OG1RF strain [26], under our conditions. After 6 days of growth on MOLP, we found that Ebp mutants and the wild-type (WT) strain exhibited similar penetration capacities, and only a slight change on the shape of the colony-print was evidenced in the absence of pili (S2A Fig). These data suggest that the bacterial migration process is mediated by an Ebp pilus-independent mechanism.

**Fig 2:**
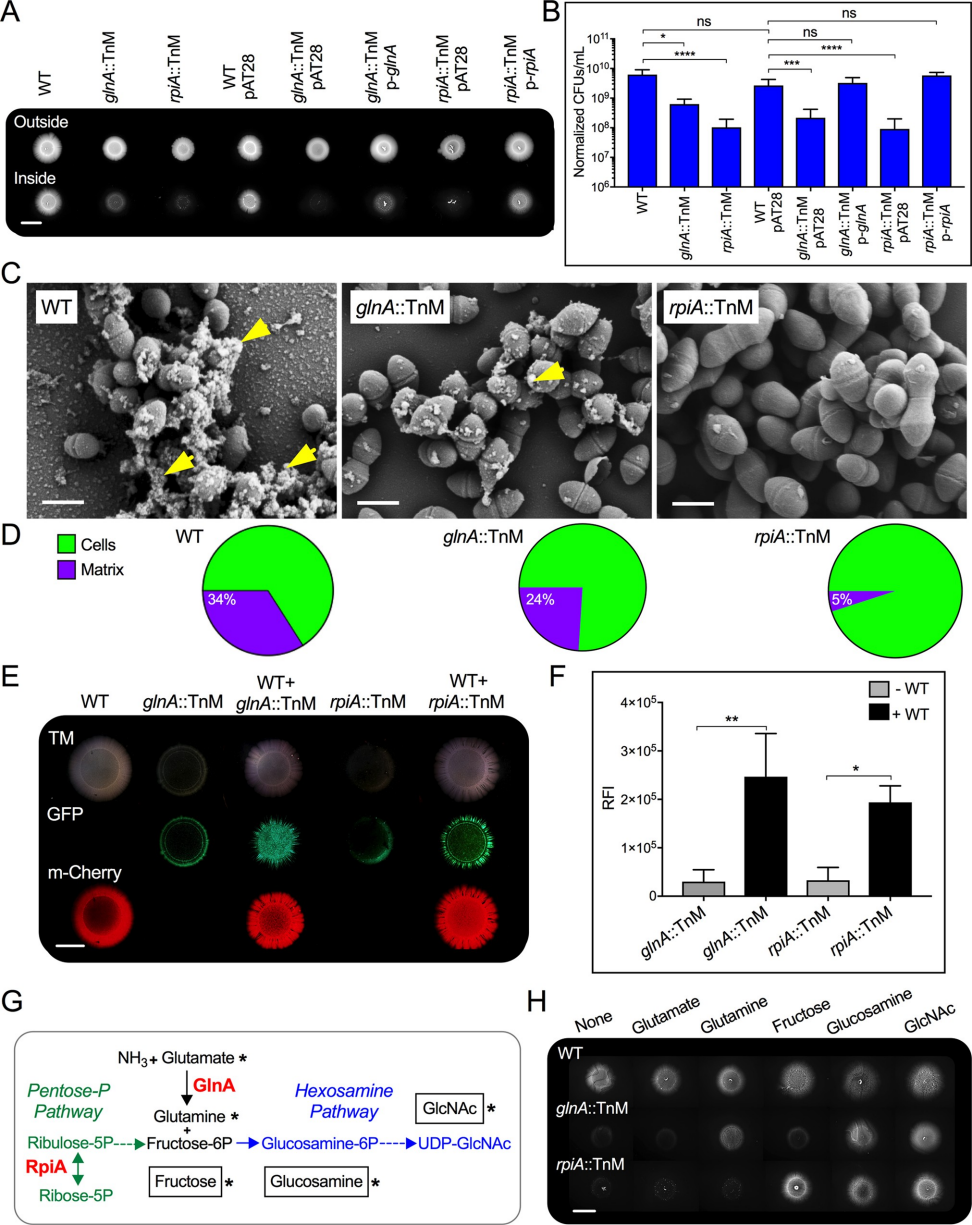
Mutations in *glnA* and *rpiA* cause defects in agar penetration that can be restored by extracellular factors or metabolic substrates. (**A** and **B**) Semisolid surface penetration by WT *E*. *faecalis* MMH594 or the Mariner transposon mutants *glnA*::TnM and *rpiA*::TnM, with and without the empty vector pAT28, or *in-trans* complemented mutants with pAT28 vector harboring their corresponding WT allele (p-*glnA* and p-*rpiA*). (**A**) Macroscopic images of colonies (outside) and invading areas of WT and mutants grown on semisolid MOLP. Scale bar: 6,000 μm. (**B**) Quantification of cells inside the semisolid surface expressed as CFUs/mL, normalized to their initial absorbance values prior to serial dilutions; (mean±SE; n = 6; *P<0.05; **P<0.01; ***P<0.001; **** *P*<0.0001 for both the one-way ANOVA and Tukey’s multiple comparison test; nonsignificant, ns). (**C**) SEM analyses of external bacterial cells. Extracellular matrix is indicated by yellow arrows (scale bar: 400 nm). (**D**) Fractions of cells and extracellular matrix found on the visible surface of SEM images of non-penetrating strains, quantified through automated image analyses. The pie chart of the non-penetrating WT is based on the same image data used in Fig 1F, outside. (**E**) Images of colony-prints of WT expressing m-Cherry grown alone or mixed with either *glnA*::TnM (WT+ *glnA*::TnM) or *rpiA*::TnM (WT+ *rpiA*::TnM) expressing GFP at a 1:2 (WT to mutant) ratio. As control, single cultures of *glnA*::TnM and *rpiA*::TnM were spot-inoculated on MOLP. Scale bar: 4,000 μm. (**F**) Relative fluorescence intensity (RFI) of the colony-prints expressing GFP generated by either mutant alone or co-cultured with WT was measured using Image J. (mean±SE; n = 3; *P<0.05; **P<0.01; one-way ANOVA with Tukey’s multiple comparison test). (**G**) Metabolic pathways depicting penetration-deficient mutants. RpiA catalyzes the reversible reaction (*double-headed arrow*) between ribose-5-phosphate (Ribose-5P) and ribulose-5-phosphate (Ribulose-5P). Through the Pentose Phosphate Pathway (*Pentose-P-Pathway*), RpiA could supply cellular pools of fructose-6-phosphate (Fructose-6P). GlnA synthesizes cellular glutamine from ammonia (NH_3_) and glutamate. Fructose-6P and glutamine could be used by the cell to form glucosamine-6-phosphate (Glucosamine-6P) that serves as intermediate to generate UDP-*N*-acetyl glucosamine (UDP-GlcNAc) in the hexosamine biosynthetic pathway (*Hexosamine pathway*). (**H**) WT or transposon mutant colonies were assessed for penetration in media without or with exogenous 5–10 mM glutamate, glutamine, fructose, glucosamine and GlcNAc. Scale bar: 6,000 μm. Substrates supplied (*asterisk*) for metabolic complementation assays (**G**).

To further understand the *E*. *faecalis* semisolid surface penetration process, we performed genetic screening of a Mariner transposon insertion library. We sought to identify mutants that developed normal colonies above the agar, but were impaired in their semisolid surface-penetration capacity. Out of approximately 6,000 mutants screened, seven were found to be defective in penetration. Five of the seven mutants identified exhibited substantial growth defects and were thus excluded from subsequent studies. Of the remaining two mutants that were unable to generate WT-like colony-prints inside the agar (Fig 2A), we determined they had transposon inserts in either the *glnA* (glutamine synthetase) or *rpiA* (ribose-5-phosphate isomerase) genes. The *glnA*::TnM, but not the *rpiA*::TnM strain, exhibited a slight growth defect in liquid MOLP (S2B Fig), but both strains formed external colonies similar in size to those of WT (Fig 2A). The external and internal numbers of bacterial cells were next determined by differential CFU analysis. No significant differences were found in the CFU counts of the *glnA*::TnM and *rpiA*::TnM mutants on the agar surface in comparison with their paternal strain (S2C Fig and S1 Table). However, inside the semisolid surface, the *rpiA*::TnM and *glnA*::TnM mutants exhibited a significant reduction in the CFU counts in comparison with their parental strain (6x10^9^; Fig 2B), consistent with the visible decrease in the colony-print generated by these two transposon mutants (Fig 2A). Genetic complementation with plasmids expressing either RpiA or GlnA correspondingly restored the invading phenotype of these mutant strains (Fig 2A and 2B). SEM analysis of external cells of six-day-old colonies revealed that while all strains exhibited diplococcal morphology, *rpiA*::TnM cells were bigger than both WT and *glnA*::TnM cells (Fig 2C). Most importantly, the extracellular matrix normally covering and connecting WT *E*. *faecalis* cells (Figs 1E and 2C) was either decreased or almost absent in *glnA*::TnM or *rpiA*::TnM mutants, respectively (Fig 2C and 2D). Together, these results indicate that GlnA and RpiA are necessary for efficient penetration of *E*. *faecalis* into semisolid surfaces, and that mutants lacking these genes also failed to produce the extracellular matrix that naturally covers the WT cells.

### Wild-type extracellular factors metabolically rescue semisolid surface penetration by *E*. *faecalis* mutants devoid of GlnA and RpiA

We next determined the molecular mechanisms by which GlnA and RpiA promote *E*. *faecalis* semisolid surface penetration. Since both enterococcal mutants unable to pass into agar failed to produce the extracellular matrix evident in the colony-prints of the parental strain (Figs 1E, 1F, 2C and 2D), we hypothesized that extracellular factors produced by WT cells could restore penetration by strains lacking GlnA and RpiA. To test this idea, WT cells expressing m-Cherry were independently mixed with GFP-labeled *glnA*::TnM or *rpiA*::TnM mutants, and colony-prints were analyzed after 6 days via fluorescence stereomicroscopy. WT invading cells formed a bright red fluorescent colony-print, whereas monocultures of *glnA*::TnM or *rpiA*::TnM showed decreased invasion capacity as evidenced by minimal GFP-derived fluorescence. However, a remarkable increase in GFP-positive invading cells was found when either mutant was co-cultured with WT cells (Fig 2E). These observations were consistent with the higher relative fluorescent intensity (RFI) observed with the colony-prints from mutants co-cultured with WT than those obtained from the monoculture area (Fig 2F). Hence, extracellular factors produced by the WT strain can restore the agar penetration defects intrinsic to the *glnA*::TnM and *rpiA*::TnM mutant cells.

GlnA and RpiA participate in key metabolic pathways (Fig 2G). GlnA plays an essential role in the metabolism of nitrogen by catalyzing the condensation of glutamate and ammonia (NH_3_) to generate glutamine [27]. RpiA catalyzes the reversible conversion of ribose-5-phosphate to ribulose-5-phosphate, a central enzymatic reaction in the pentose phosphate pathway [28]. We hypothesized that the metabolic functions of GlnA and RpiA may converge in the hexosamine biosynthetic pathway, where the glutamine produced by GlnA, together with fructose-6-phosphate generated from metabolites of the pentose phosphate pathway, could promote the formation of glucosamine-6-phosphate [29, 30]. We further postulated that decreased ability to penetrate MOLP by *glnA*::TnM and *rpiA*::TnM mutants could be a consequence of alterations in the levels of intracellular hexosamine biosynthetic pathway metabolites (Fig 2G). To test these hypotheses, we performed a metabolic complementation assay by supplementing exogenous substrates related to this pathway. Semisolid surface penetration by WT cells was not altered by addition of any of the substrates to the medium (Fig 2H). However, exogenous glutamine, glucosamine and GlcNAc, but not glutamate or fructose, rescued the defective penetration phenotype of *glnA*::TnM cells. In addition, penetration of *rpiA*::TnM mutants was restored by fructose, glucosamine or GlcNAc supplementation (Fig 2H). These data suggest that low availability of cellular fructose-6-phosphate (in *rpiA*::TnM) or glutamine (in *glnA*::TnM) compromises the hexosamine biosynthetic pathway and consequently, decreases the cellular levels of products of this pathway such as glucosamine-6P and UDP-GlcNAc that are required for enterococcal migration into semisolid MOLP.

### GlcNAc-derived exopolysaccharides are necessary for *E*. *faecalis* semisolid surface penetration

**Fig 3:**
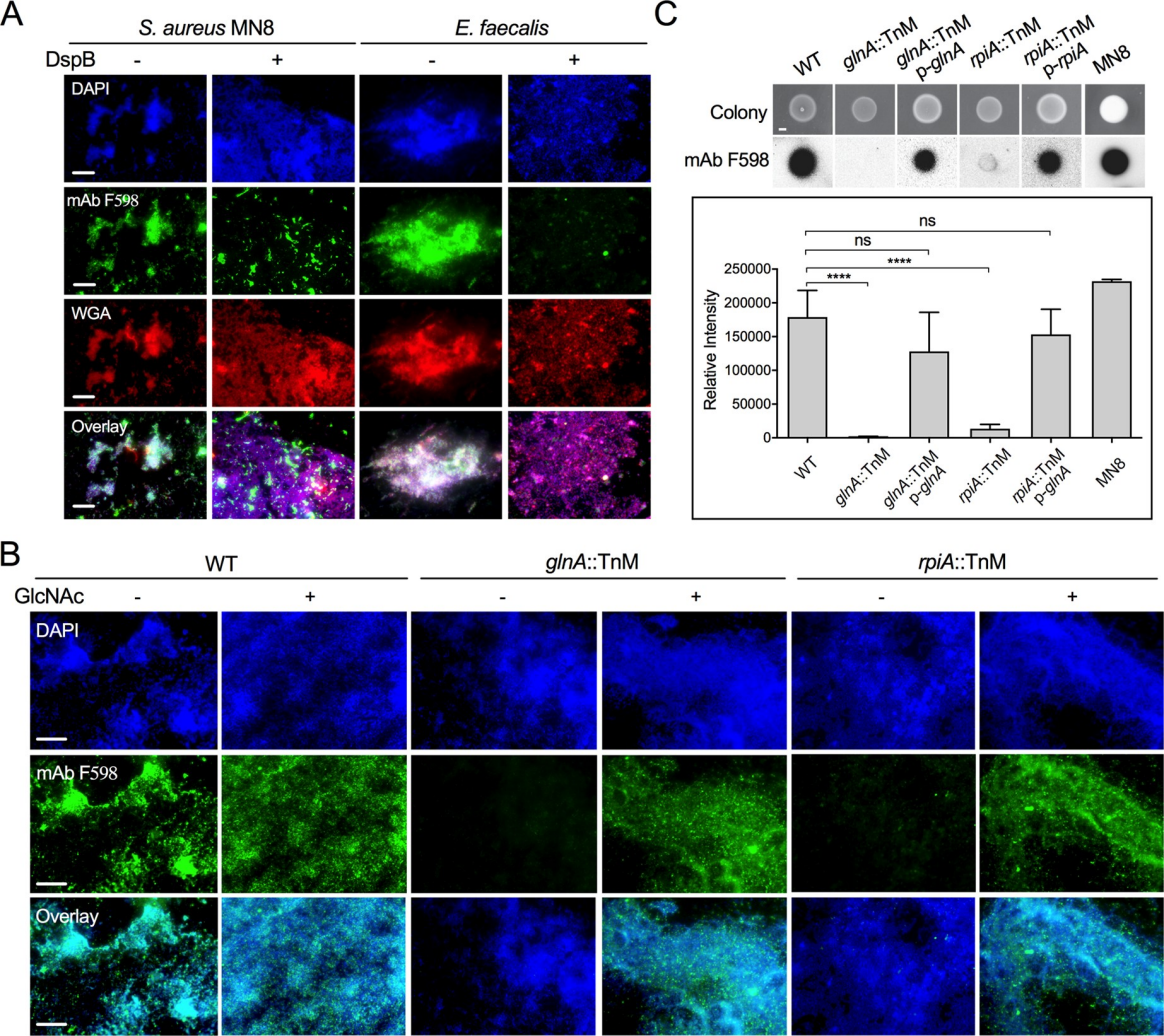
polyGlcNAc-containing exopolysaccharides are necessary for *E*. *faecalis* semisolid media penetration. (**A** and **B**) Immunofluorescence analysis of cells stained with either Texas red-conjugated WGA or mAb F598, which specifically binds to β-1,6-linked GlcNAc polysaccharides. To visualize antibody binding to GlcNAc-containing polymers, cells were reacted with a secondary anti-human IgG conjugated to Alexa Fluor-488 (green fluorescence). DAPI was used to stain bacterial DNA (blue fluorescence). (**A**) *Staphylococcus aureus* MN8 (positive control) and *E*. *faecalis* MMH594 non-penetrating cells from MOLP-grown colonies were treated with or without 300 μg/mL Dispersin B (DspB), an enzyme that degrades β-1,6 bonds. (**B**) Cells of *E*. *faecalis* WT and transposon mutants grown on MOLP for 6 days with (+) or without (-) 10 mM GlcNAc were tested for binding to mAb F598 as described above. (**C**) Colony hybridization blot (*top panel*) of 24 hours-grown-colonies of WT and *glnA*::TnM and *rpiA*::TnM alone or harboring the pAT28 vector containing their corresponding WT allele (p-*glnA* and p-*rpiA*). The relative intensity obtained upon incubation with mAb F598 was calculated for each colony using Image J (*lower panel*); (mean±SE; n = 8; ****P<0.0001 for both the one-way ANOVA and Turkey’s multiple comparison test). Scale bars (**A** and **B**): 20 μm and (**C**): 1000 μm.

We postulated that products derived from UDP-GlcNAc could mediate agar penetration by *E*. *faecalis*. Interestingly, we observed that *E*. *faecalis* readily produced extracellular GlcNAc-containing products, as evidenced by staining with wheat germ agglutinin (WGA; Fig 3A) that binds to GlcNAc residues [31]. *S*. *aureus* MN8, a bacterial strain that has been shown to secrete GlcNAc-derived polymers[8, 32], was used in this assay as a positive control (Fig 3A).

Since UDP-GlcNAc is a common precursor for the synthesis of bacterial cell walls and some polyGlcNAc exopolysaccharides, such as PNAG (PIA) [9], we hypothesized that polyGlcNAc-containing exopolysaccharides could mediate enterococcal semisolid surface penetration. To test this, we examined the surface of enterococcal colonies grown on MOLP by immunofluorescence using the monoclonal antibody (mAb) F598, which specifically recognizes β-1,6-linked polyGlcNAc polymers [8, 11]. We detected polyGlcNAc-containing polymers on both the WT enterococcal cell surface and the positive control *S*. *aureus* (Fig 3A). The staining specificity was confirmed with an *S*. *aureus* strain (Δ*ica*) unable to produce the polyGlcNAc polymer, PNAG (PIA) [32] (S3A Fig). Further validating these results, the presence of these polymers was decreased and severely mislocalized in both WT *E*. *faecalis* and the positive control *S*. *aureus* MN8 upon treatment with Dispersin B (DspB; Fig 3A), an enzyme that specifically cleaves the β-1, 6 linkage of glucosamine and depolymerizes PNAG (PIA) [33]. DspB treatment did not affect the binding of WGA to any WT strain (Fig 3A), suggesting that this lectin may react with additional cellular components different from β-1,6-linked GlcNAc polymers. Indeed, WGA has been shown to detect not only GlcNAc residues, but also β-1,4-linked GlcNAc oligomers [31] such as the exposed-GlcNAc residues of the peptidoglycan layer of Gram (+) bacteria [34]. This observation was further confirmed by the remaining positive WGA signal found in the PNAG (PIA)-deficient *S*. *aureus* mutant Δ*ica* (S3A Fig). In contrast to WT *E*. *faecalis*, polyGlcNAc-containing polymers were not detected in *glnA*::TnM or *rpiA*::TnM mutants stained with mAb F598 (Fig 3B). Strikingly, metabolic complementation using GlcNAc-supplemented media restored polyGlcNAc-derived polysaccharide production (Fig 3B) and semisolid surface penetration (Fig 2H) in these two mutant strains.

We performed colony immunoblot assays to define the localization of polyGlcNAc-containing polysaccharides. Non-lysed *E*. *faecalis* cells from colonies grown on MOLP were transferred onto nitrocellulose membranes and incubated with mAb F598 to detect polymer production. Consistent with our microscopy results, we only observed a strong signal in the WT strain but not in *glnA*::TnM and *rpiA*::TnM mutants. Complementation by either addition of exogenous GlcNAc to the media (S3B and S3C Fig) or with plasmids expressing either RpiA or GlnA correspondingly (Fig 3C) restored extracellular polyGlcNAc-derived polysaccharide production. Similarly, the control *S*. *aureus* MN8 also demonstrated positive detection in these analysis (Fig 3C; and S3C Fig).

To further characterize the exopolysaccharides produced by *E*. *faecalis* grown on semisolid surfaces, we used calcofluor white (CFW), a fluorescent dye known to bind surface fibrillar exopolysaccharides harboring either β-1,3 or β-1,4 linkages such as cellulose and chitin [35–37]. In contrast to the CFW positive binding observed with *Candida albicans* colonies, a strain known to synthesize chitin (a β-1,4-linked oligosaccharide) [38], *E*. *faecalis* colonies did not produce CFW-reactive exopolysaccharide under our conditions. Similarly, the negative control *Escherichia coli* DH5α [39], did not exhibit any fluorescence with CFW in the culture media (S3D Fig). Taken together, these data suggest that *E*. *faecalis* produces β-1,6-linked GlcNAc-containing polysaccharides that are extracellularly localized and necessary for agar penetration capacity.

### EpaX is required for exopolysaccharide-mediated semisolid surface penetration

**Fig 4:**
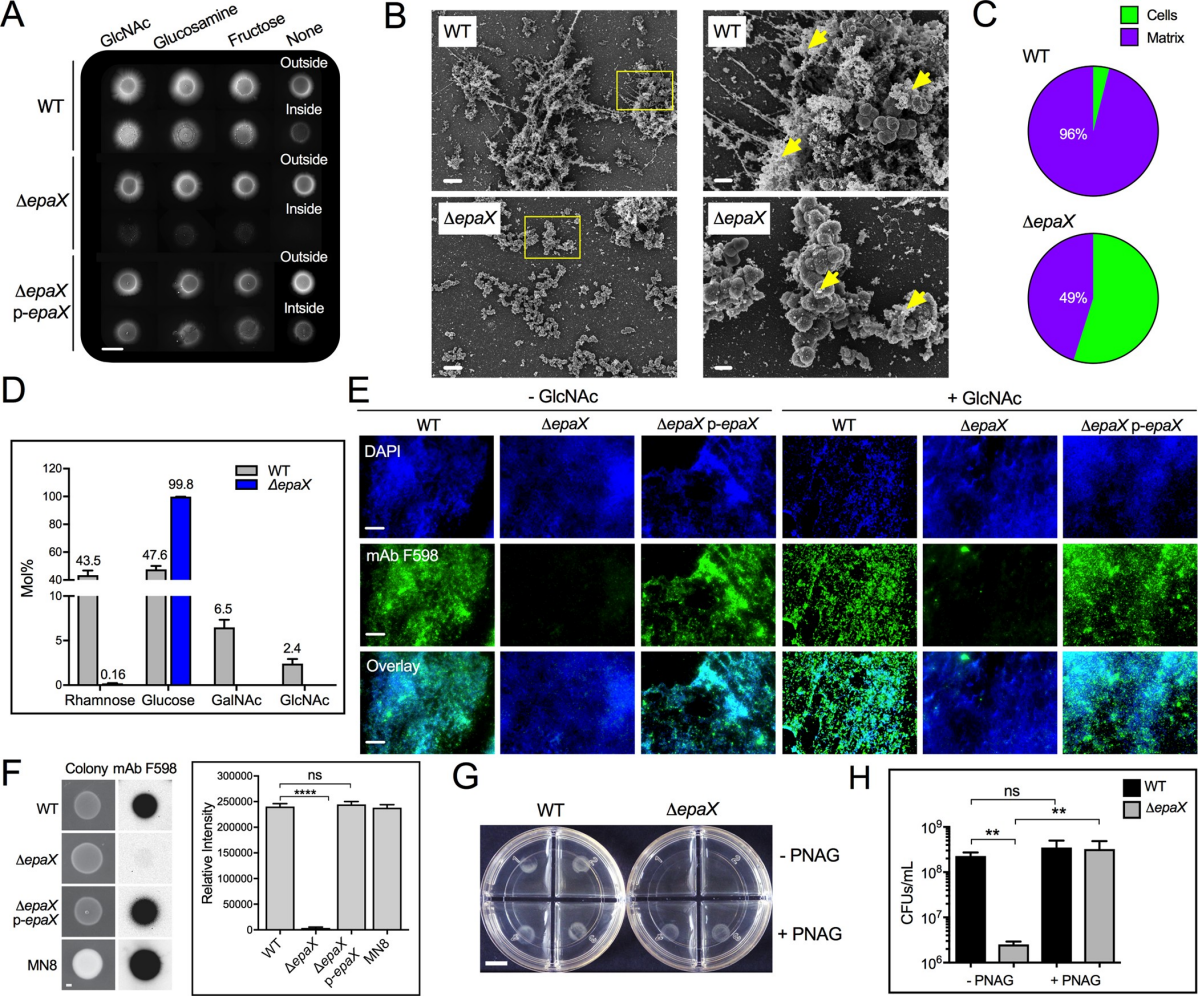
EpaX is necessary for *E*. *faecalis* semisolid surface penetration and synthesis of polyGlcNAc-containing polysaccharides. (**A**—**H**) Penetration analysis for *E*. *faecalis* VE14089 (closely-related to MMH594) and the EpaX-deficient mutant (Δ*epaX*) (**A**) Invasion was tested for mutant and its parental strain with (*right panel*) or without exogenous GlcNAc, glucosamine or fructose. Complementation in-trans of *epaX* WT allele restored invasion (p-*epaX*). Scale bar: 6,000 μm. (**B**) SEM images of external cells at (*left*) low magnification (*scale bar*: 5 μm). Yellow squared areas were imaged at higher magnifications (*right*; scale bar: 1 μm), where extracellular matrix is indicated (*yellow arrow head*). (**C**) Fractions of cells and extracellular matrix found on the visible surface of SEM images of non-penetrating WT and mutant strains. (mean±SE; n = 3; *P<0.05; two-tailed unpaired *t*–test) (**D**) Glycosyl composition of polysaccharides extracted from liquid cultures of WT and Δ*epaX* grown to exponential phase in MOLP broth (mean±SE; n = 3). (**E**) Fluorescence microscopy images of external cells from MOLP-grown colonies incubated with mAb F598 to visualize GlcNAc-containing exopolysaccharides (green fluorescence). DAPI was used to stain bacterial DNA (blue fluorescence). Scale bar: 20 μm. (**F**) Immunoblot (*left panel*) of 24-hour-grown colonies. The relative intensity obtained upon incubation with mAb F598 was calculated for each colony using Image J, *right panel*; (mean±SE; n = 8; ****P<0.0001 for both one-way ANOVA and Turkey’s multiple comparison test). Scale bar: 1000 μm. (**G**) Semisolid media penetration of WT and Δ*epaX* grown with and without 200 μM PNAG purified from *S*. *aureus* MN8. Scale bar: 5,000 μm. (**H**) Quantification of invasion was expressed as CFUs/mL (mean±SE; n = 4; **P<0.01 for both the one-way ANOVA and Tukey’s multiple comparison test).

In *S*. *aureus*, the synthesis of polyGlcNAc polymers, such as PNAG (PIA), depends on the expression of biosynthetic enzymes encoded by the *icaADBC* operon [40–42]. IcaA is a glycosyltransferase that uses UDP-GlcNAc as a substrate [40], and IcaB is responsible for the deacetylation of PNAG (PIA) [43]. Since *in silico* analyses revealed that *E*. *faecalis* does not have homologs of these genes, we used Nanostring technology [44, 45] to identify potential glycosyltransferase genes that could be involved in synthesizing polyGlcNAc-containing polysaccharides that mediate *E*. *faecalis* agar penetration. Transcript levels of genes encoding putative glycosyltransferases were determined in cell lysates from *E*. *faecalis* colonies undergoing agar penetration, normalizing gene expression to multiple independent housekeeping genes (S2 Table). Several genes demonstrated significant expression changes during the semisolid surface entering process (S4A Fig). We focused on EF2170 (*epaX*) because its transcript levels were markedly elevated in cells that entered the agar, compared with non-penetrating cells on the surface (S4A Fig). To determine the role of EpaX in enterococcal semisolid surface penetration, we tested a strain harboring mutations in the *epaX* gene (EF2170) [46]. This mutant had no apparent growth defects under our conditions (Fig 4A and S4B Fig), but demonstrated a substantial defect in agar penetration, which was corrected upon genetic complementation with a plasmid expressing EpaX (Fig 4A). Further confirming these results, deletion of *epaX* in a closely-related *E*. *faecalis* strain (MMH594) also resulted in attenuated semisolid surface invasion (S4C Fig). Similarly to *glnA*::TnM and *rpiA*::TnM strains, SEM analysis of external *E*. *faecalis* cells in six-day-old colonies revealed that Δ*epaX* showed a profound reduction in the extracellular matrix normally covering and connecting its WT counterpart (Fig 4B and 4C).

To further characterize the role of EpaX in the synthesis of polysaccharides under our penetration conditions, we used polyacrylamide gel electrophoresis with subsequent alcian blue and silver nitrate staining to analyze the polysaccharide content of WT and Δ*epaX* extracted from colonies grown on MOLP. Consistent with previous results [46], a band disappeared in the Δ*epaX* strain, which was restored upon genetic complementation (S4D Fig), suggesting drastic changes in the oligosaccharide composition between these strains. Indeed, further analysis using acid methanolysis combined with gas chromatography-mass spectrometry (GC-MS) determined that loss of EpaX severely altered the glycosyl composition of *E*. *faecalis* (Fig 4D). Specifically, Δ*epaX* stains demonstrated increased glucose content that was accompanied by a profound reduction in rhamnose, GalNAc and GlcNAc, compared with their parental strain (Fig 4D). These data suggested that EpaX might be required for the synthesis of GlcNAc-containing exopolymers that are necessary for optimal *E*. *faecalis* semisolid surface penetration. To test this idea, we performed immunofluorescence analyses using mAb F598. Notably, E. faecalis Δ*epaX* was not recognized by the antibody (Fig 4E and S4E Fig), but this defect could be corrected upon genetic complementation with a plasmid encoding EpaX (Fig 4E). The binding of WGA to *E*. *faecalis* was unaltered in the absence of EpaX (S4E Fig), suggesting that this putative glycosyl transferase is necessary to generate β-1,6-polyGlcNAc-containing polymers, but not to synthesize other GlcNAc-containing cellular components or polysaccharides detected by the lectin. Moreover, colony immunoblot assays further demonstrated that polyGlcNAc-containing exopolysaccharides were detected only in colonies from strains with a functional EpaX (Fig 4F). To define whether EpaX operates upstream or downstream of GlnA and RpiA, we tested if exogenous fructose, glucosamine or GlcNAc could rescue the defective invasive phenotype of Δ*epaX*, as previously observed in *glnA*::TnM and *rpiA*::TnM mutants (Fig 2H). The WT parental strain formed bigger colonies and penetrated more efficiently when fructose, glucosamine and GlcNAc were supplemented. However, none of these substrates rescued penetration in Δ*epaX* strains (Fig 4A and S4C Fig). Supplementation of exogenous GlcNAc also failed to restore polyGlcNAc-containing polymer production by the Δ*epaX* strain (Fig 4E). Strikingly, however, exogenous addition of exogenous purified PNAG (PIA) from *S*. *aureus* MN8 fully restored invasion by Δ*epaX* strains (Fig 4G). CFUs quantification of invading cells confirmed that *S*. *aureus* MN8-derived PNAG (PIA) rescued the attenuated invasive phenotype observed in Δ*epaX* (Fig 4H). No major bacterial growth defects were observed upon PNAG (PIA) addition to semisolid media (S4F and S4G Fig). Together, these results demonstrate that EpaX functions downstream of GlnA and RpiA to drive β-1,6-linked polyGlcNAc polymer-mediated surface penetration in *E*. *faecalis*.

### PolyGlcNAc-containing polymers are required for *E*. *faecalis* translocation

**Fig 5:**
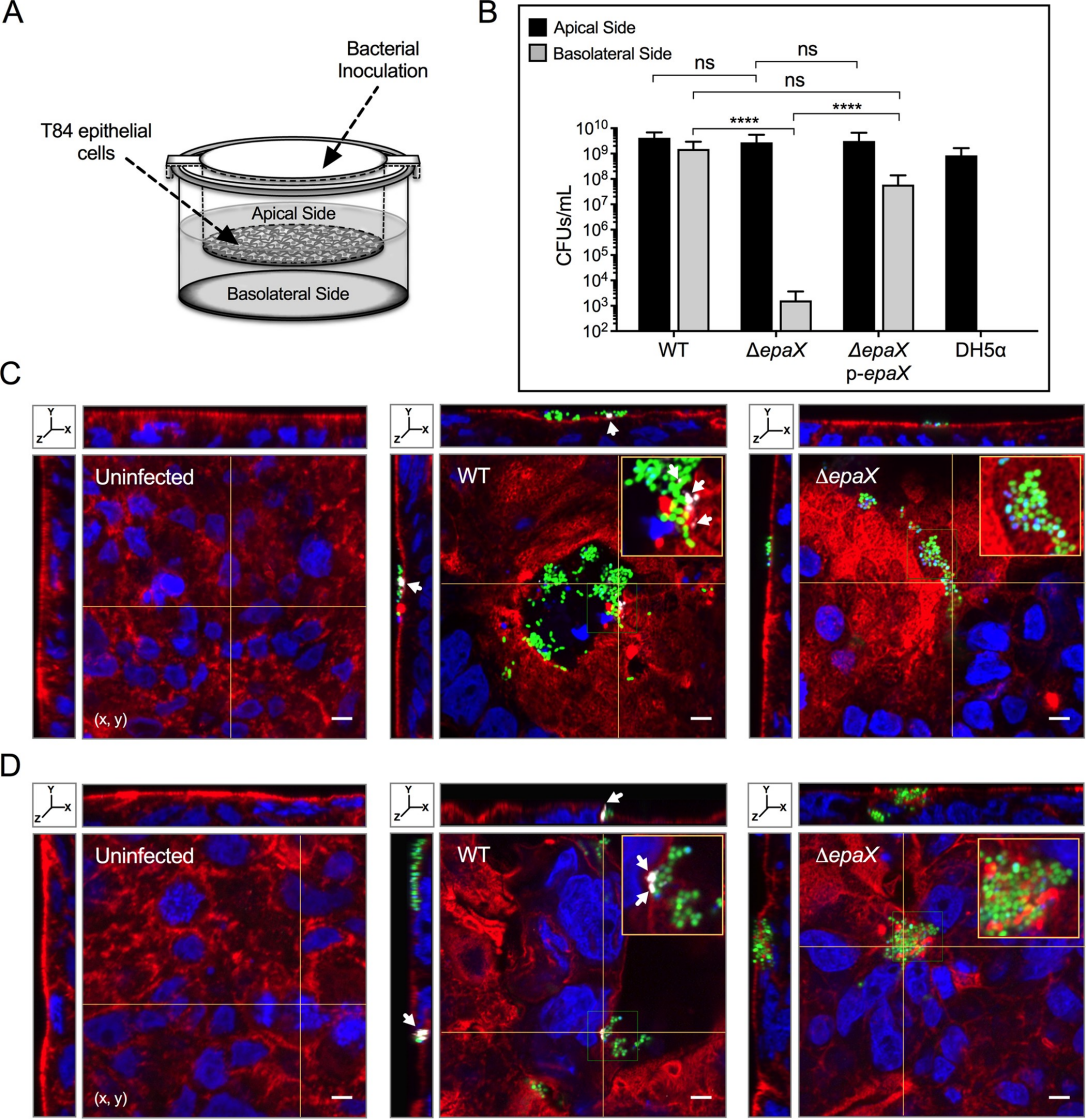
*E*. *faecalis* strains devoid of polyGlcNAc-containing polysaccharides demonstrate defective translocation through human intestinal epithelial cell monolayers. (**A**) Scheme of the two-chamber translocation system. *E*. *faecalis* VE14089 WT and derived strains inoculation occurred at the apical side. T84 human intestinal epithelial cell monolayers grown on filters separate the apical side from the lower chamber. (**B**) Colony forming units (CFUs/mL) of viable bacterial cells that did not pass through the monolayer (apical side) or translocated to the basolateral side after 8 hours of incubation. *Escherichia coli* DH5α was used as a negative control (mean±SE; n = 5; ****P <0.0001 for both the one-way ANOVA and Tukey’s multiple comparison test). (**C** and **D**) 3D reconstruction of confocal immunofluorescence images (x, y, z) of T84 human cell monolayers uninfected (*left*) or infected for 2 (**C**) or 6 (**D**) hours with WT (*center*) or Δ*epaX* (*right*) constitutively expressing GFP (green fluorescence). Alexa Fluor-594 coupled phalloidin and DAPI were used to stain the epithelial cell actin (red fluorescence) and nucleus (blue fluorescence), respectively. To visualize polyGlcNAc-containing polymers, epithelial cells and enterococci co-cultures were treated with mAb F598 antibody and reacted with the antihuman IgG conjugated to Alexa Fluor-647 (gray fluorescence; *white arrows*). The yellow lines mark the intersection point were the x, z (*top*) and y, z (*left*) orthogonal views of reconstructed Z-sections were taken. Scale bar: 5 μm. The orange square represents a 2X-magnified view of the selected area in x, y planes (green square).

*E*. *faecalis* has the potential to translocate from the gastrointestinal tract to the blood stream [47], most likely via a paracellular mechanism that allows the bacterium to move through epithelial cell monolayers [48]. We hypothesized that *E*. *faecalis* mutants defective in biosynthesis of polyGlcNAc-containing polymers and semisolid surface penetration would also be altered for translocation through intestinal epithelial barriers. To this end, we used a previously described two-chamber transcytosis system [49, 50] where translocation is evaluated by determining the bacterial number (as CFUs) capable of passing from the apical side through T84 human intestinal epithelial cell monolayers, to the basolateral side of the chamber (Fig 5A). We found that 8 hours post-infection, the integrity of inoculated and non-inoculated monolayers exhibited transepithelial resistance values similar (~8,900 Ω/cm^2^) to those obtained prior to bacterial inoculation (~8,300 Ω/cm^2^), thus indicating that the T84 cell monolayers remained mostly intact throughout the experiment. All strains evaluated reached approximately 10^8^−10^9^ CFUs/mL in the apical side of all the wells tested (Fig 5B, S5A and S5B Fig). Consistent with previous reports [49, 50], the negative control *E*. *coli* DH5α was not detected in the lower chamber of any of the inserts analyzed (Fig 5B, S5A and S5B Fig), but WT *E*. *faecalis*, in sharp contrast, demonstrated a remarkable capacity to translocate in this assay. When *E*. *faecalis* mutant strains were evaluated, reduced numbers of *rpiA*::TnM cells (~5x10^2^ CFUs/mL) were detected in the basolateral section in comparison with its parental WT strain (~6x10^7^ CFUs/mL; S5A Fig). Interestingly, the *glnA*::TnM mutant did not show a significant decrease in translocation, likely due to metabolic complementation by exogenous glutamine present in the translocation culture medium (S5A Fig). Indeed, removing glutamine from the system drastically attenuated the translocation capacity of *glnA*::TnM but not WT cells (~4x10^2^ vs. ~1x10^8^ CFUs/mL, respectively; S5B Fig). Similarly, while WT *E*. *faecalis* moved efficiently through epithelial cell monolayers, Δ*epaX* demonstrated a significant decrease in translocation (~2x10^9^ and ~1x10^3^ CFUs/mL for WT and mutant, respectively; Fig 5B). Of note, genetic complementation of *rpiA*::TnM, *glnA*::TnM or Δ*epaX* cells restored the ability of each corresponding mutant strain to translocate (Fig 5B, S5A and S5B Fig). As control in our assays, we used a Δ*epaB* deletion strain unable to produce the glycosyl transferase EpaB (Orfde4), a protein previously shown to be necessary for efficient *E*. *faecalis* translocation through human epithelial cell monolayers [6, 49]. Surprisingly, under our conditions, the translocation ability of the Δ*epaB* mutant was similar to its parental strain (S6A Fig). In addition, Δ*epaB* was capable of producing polyGlcNAc-containing polymers at similar levels as the WT strain (S6B and S6C Fig), and exhibited normal capacity to penetrate semisolid agar (S6D Fig). These data suggest that, under our experimental conditions, the synthesis of polyGlcNAc-containing polymers is sufficient to enable surface penetration by strains devoid of EpaB.

To further characterize *E*. *faecalis* translocation, T84 human intestinal epithelial cell monolayers were infected with GFP-labeled *E*. *faecalis* parental and mutant strains. Immunofluorescence analyses were performed by reacting each sample with phalloidin, DAPI and mAb F598 to visualize the actin cytoskeleton, nuclei (and bacterial DNA) and polyGlcNAc-containing polymers, respectively. Laser scanning confocal microscopic analyses of stained samples were carried out to localize bacteria within the T84 monolayers. After two hours of infection, we observed that enterococci were frequently co-localized with actin-rich areas (Fig 5C and S5C and S5D Fig). Moreover, the orthogonal views showed that bacterial aggregates concentrated on the top of the monolayers where parental strains (WT) formed surface invaginations, in comparison with the smooth surface of intact T84 human intestinal epithelial monolayers (Fig 5C, S5C and S5D Fig), hence suggesting that WT strains alter the actin cytoskeleton during the translocation process. These surface perturbations were observed to a lesser extent in monolayers infected with either Δ*epaX* (Fig 5C), *rpiA*::TnM (S5C Fig) or *glnA*::TnM mutants (S5D Fig). Importantly, polyGlcNAc-containing polysaccharides were detected around WT cell aggregates, but not in any of the mutants tested (Fig 5D, S5C and S5D Fig). These polysaccharides were frequently found to cover (or be adjacent to) bacterial cells, and their presence was visualized even after 6 hours post-infection (Fig 5D). Interestingly, we only observed surface openings of the epithelial barrier upon incubation with WT strains, suggesting that host cell lysis is caused by *E*. *faecalis* during the translocation process (Fig 5C and 5D and S5C and S5D Fig).

Taken together, our data reveal that *E*. *faecalis* utilizes the RpiA-GlnA-EpaX axis to generate β-1,6-linked polyGlcNAc-containing exopolysaccharides necessary for optimal migration into semisolid surfaces and for efficient paracellular translocation through human epithelial cell monolayers.

## Discussion

**Fig 6:**
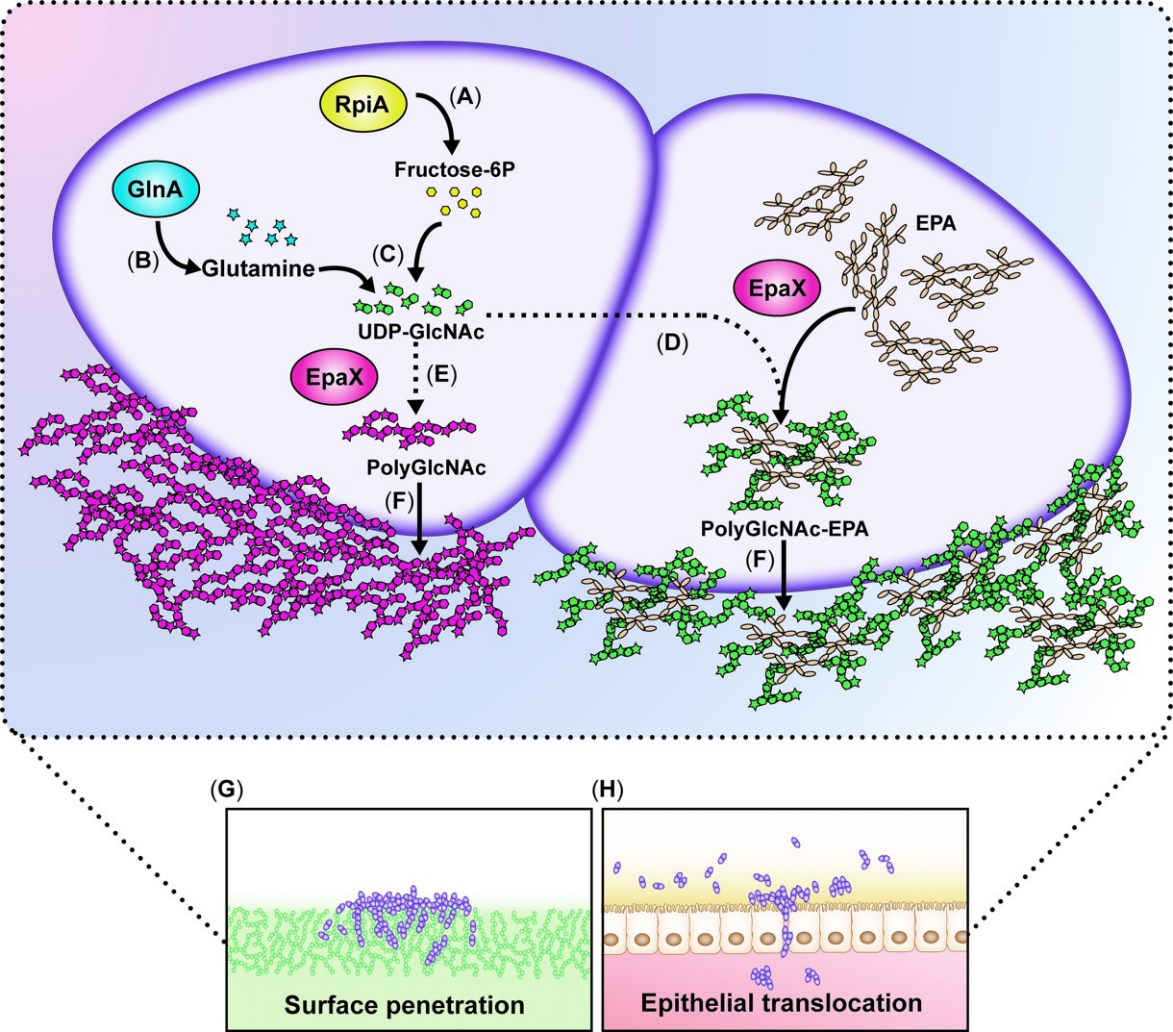
Proposed model for *E*. *faecalis* surface penetration using polyGlcNAc-containing polymers. (**A**) RpiA is required to generate ribulose-5-phosphate, a substrate that could be converted into fructose 6-phosphate (Fructose-6P) in the non-oxidative phase of the pentose phosphate pathway. (**B**) GlnA generates glutamine from glutamate and ammonia. (**C**) Cellular fructose-6P and glutamine converge in the hexosamine biosynthetic pathway to ultimately generate UDP-GlcNAc, which is used by EpaX to synthesize an enterococcal polyGlcNAc-containing polymer, which could be a decoration of the rhamnopolysaccharide EPA (**D**) or a yet unknown polymer (**E**). These polyGlcNAc-containing polysaccharides are extracellularly localized (**F**) and promote the movement and penetration of *E*. *faecalis* into semisolid surfaces (**G**) or through human epithelial cell monolayers (**H**). In the absence of a functional RpiA or GlnA, the levels of UDP-GlcNAc are not sufficient to promote polysaccharide synthesis, thus blocking efficient surface penetration and/or translocation.

In this study, we uncovered molecular pathways and metabolic mediators that endow *E*. *faecalis* with the capacity to move into semisolid surfaces and translocate through human epithelial cell barriers (proposed model; Fig 6).

Exopolysaccharides have been well characterized as prominent components of the extracellular matrices of surface-associated multicellular communities termed biofilms [51–53]. Our study reveals a new role for polyGlcNAc-containing extracellular polysaccharides as key mediators of *E*. *faecalis* migration traits. These exopolysaccharides may operate as a “glue” that holds cells together [52, 54, 55] while promoting the formation of matrix-encased multicellular aggregates during enterococcal migratory behavior. Indeed, it has been proposed that polyGlcNAc polymers facilitate intercellular adhesion by bridging electrostatic interactions between cells surfaces [56]. Alternatively, or in addition, polyGlcNAc-containing hydrated exopolysaccharides may help *E*. *faecalis* to spread in a manner similar to that found in *Proteus mirabilis*, which secretes polysaccharides that create a fluidic environment promoting movement on surfaces with low moisture [57]. Furthermore, in *B*. *subtilis*, a PNAG (PIA)-defective strain was shown to lose its hydrophobic or nonwetting surface characteristics [58], indicating that polyGlcNAc polymers provide a means to shape the external environment in a manner amenable to bacterial movement or penetration. Additional biophysical and chemical analyses are thus warranted to comprehensively understand how these glycopolymers promote surface penetration by *E*. *faecalis*.

Our study unearths new metabolic factors mediating enterococcal surface penetration. The first one is GlnA, which plays an essential function in the generation of glutamine [27] that is used as a constituent of proteins and a nitrogen donor for many biosynthetic reactions [59, 60]. The second factor is RpiA, which catalyzes a central enzymatic reaction in the pentose phosphate pathway that is a major route of intermediary carbohydrate metabolism. RpiA is also involved in the generation of lipopolysaccharide components in Gram-negative bacteria [28]. Our results indicate that the metabolic functions of GlnA and RpiA converge in the hexosamine biosynthetic pathway to generate the UDP-GlcNAc necessary to produce polyGlcNAc-containing polymers such as PNAG (PIA) [61]. Based on our genetic and metabolic supplementation experiments, we propose that the hexosamine biosynthetic pathway and the pentose phosphate pathway supply metabolic substrates that serve as precursors for synthesizing enterococcal polyGlcNAc-containing polymers (see proposed model, Fig 6). A link between the pentose phosphate pathway and polysaccharide synthesis was previously described in bacteria. Somerville and colleagues reported that the *S*. *aureus* transcriptional regulator RpiR, which is known to control *rpiA* expression, also acts as a sugar-responsive regulator that modulates polysaccharide synthesis in response to metabolite concentrations [62].

The biosynthesis of polysaccharides first occurs in the cytoplasm, and the repeating units are then assembled and exported to the surface. This process involves several key enzymes including glycosyltransferases that mobilize sugar units [63]. Our study emphasizes a major role for the putative glycosyltransferase EpaX in *E*. *faecalis* physiology, as this enzyme was pivotal for semisolid surface penetration and paracellular translocation. Supporting this concept, Rigottier-Gois and colleagues had demonstrated that EpaX is a major determinant of *E*. *faecalis* intestinal colonization in mice [46]. Of note, the penetration-defective phenotype of Δ*epaX* could not be complemented by exogenous GlcNAc, indicating that EpaX is required for synthesis of the polyGlcNAc structure needed for optimal surface migration. Our data also show that EpaX acts downstream of RpiA and GlnA, which explains why only the addition of exogenous PNAG (a polyGlcNAc polymer) could rescue the penetration-defective phenotype of Δ*epaX* strains. Consistent with the notion that GlcNAc-derived polysaccharides are necessary for semisolid surface penetration, we demonstrated that *epaX* mutants do not produce detectable amounts polyGlcNAc-containing exopolymers. Interestingly, bioinformatic analysis using the Protein Homology/AnalogY Recognition Engine (Phyre^2^) [64] indicated that EpaX has 100% similarity across its predicted secondary structure to glycosyltransferases such as *N*-acetylgalactosamyltransferases. Furthermore, analysis using the conserved domain architecture retrieval tool (CDART) revealed that EpaX also has similar domain architecture to YdaM, a putative glycosyltransferase shown to be required for exopolysaccharide synthesis in *Bacillis subtilis* [65]. However, the function of EpaX remains elusive, and its activity might have an epistatic relationship with other factors required for the production or cell surface display of polyGlcNAc-containing polymers. Recent studies proposed that *epaX* deletion alters the synthesis of the rhamnopolysaccharide EPA in *E*. *faecalis* by compromising the decoration of these polymers with galactose and/or GalNAc. Therefore, it was suggested that EpaX functions as a GalNAc transferase [46]. Our findings indicate that, under our conditions, the absence of EpaX in *E*. *faecalis* not only dramatically decreases the levels of GalNAc-, but also of rhamnose- and GlcNAc-containing oligosaccharides. Though rhamnose was not detected in the polysaccharides produced by Δ*epaB* using GC-MS analysis [6], we found that *E*. *faecalis* lacking EpaB was still able to penetrate and generate polyGlcNAc-containing polymers, suggesting that the presence of GlcNAc, but not rhamnose, in *E*. *faecalis* exopolymers is necessary for optimal penetration into semisolid surfaces. While the structure of *E*. *faecalis* EPA has not been elucidated, similar polysaccharides with branching structures composed by other oligosaccharides bound to GlcNAc or terminal β-linked GlcNAc side chains have been evidenced in other Gram-positive bacteria [66]. Indeed, our results using DspB demonstrated the presence of β-1,6 glycosidic bonds within the structure of *E*. *faecalis* polyGlcNAc-containing exopolysaccharides. However, the precise nature of the polymer involved in *E*. *faecalis* semisolid surface and epithelial barrier penetration has not yet been elucidated by purification and chemical analyses. Indeed, either EPA or another polysaccharide yet to be identified might mediate the penetration process. Future analyses to determine the structure of *E*. *faecalis* polyGlcNAc-containing exopolymers, and their link with EPA, will hence be of significant interest.

*E*. *faecalis* is a leading cause of nosocomial infections world-wide [67]. It has been shown that *E*. *faecalis* can translocate across mouse and rat intestinal tracts to reach other body sites [68, 69]. Most recently, Krueger et al. reported that after feeding mice with antibiotics, *E*. *faecalis* could be found in the liver, spleen, and mesenteric lymph nodes [70, 71]. PolyGlcNAc-like polysaccharides might mediate these processes by promoting enterococcal translocation across the intestinal epithelium. Interestingly, *E*. *faecalis* has been shown to form microcolonies surrounded by an extracellular matrix that not only covers the bacterial cells, but also extends into the intestinal space between cell clusters [72]. In line with our observations during semisolid surface invasion and epithelial barrier assays, Peng and collaborators described that *E*. *faecalis* formed cellular aggregates that localized with the actin cytoskeleton during the process of translocation [48]. Our findings therefore uncover that production of polyGlcNAc-containing exopolysaccharides is a mechanism that enables non-motile *E*. *faecalis* to penetrate semisolid surfaces and cross human intestinal epithelial cell monolayers.

## Materials and methods

### Bacterial strains, media and culture conditions

**S3** Table describes all strains and plasmids used in this study [6, 10, 26, 32, 46, 73–83] *E*. *faecalis* was cultured overnight at 37°C in Tryptic Soy Broth (TSB) with 0.25% Glucose (Becton Dickinson) under shaking conditions, unless indicated otherwise. *E*. *coli* strains were cultured in Lysogeny Broth (LB). Antibiotics were added to the medium when appropriate as follows: Chloramphenicol 10 μg/mL, spectinomycin 150 μg/mL or ampicillin 100 μg/mL for *E*. *coli*. Either tetracycline 15 μg/ml, chloramphenicol 10–15 μg/mL or spectinomycin 750 μg/mL for *E*. *faecalis* strains when specified. All chemicals were purchased from Sigma-Aldrich unless stated otherwise.

### Semisolid surface penetration assay

2 μL of TSB-grown *E*. *faecalis* overnight cultures were inoculated onto modified medium optimal for lipopeptide production (MOLP) [24], containing 30 g/L peptone, 7 g/L yeast extract, 1.0 mM MgSO_4_, 25 μM MnSO_4_, 25 μM FeCl_2_, 0.001 mg/L CuSO_4_, 0.004 mg/L Na_2_MoO_4_, 0.002 mg/L KI, 5 μM ZnSO_4_.7H_2_O, 0.001 mg/L H_3_BO_3_, 25 mM potassium phosphate buffer (pH 7), 125 mM MOPS (morpholinepropanesulfonic acid; pH 7) and 10 g/L agar (Becton Dickinson). Saline solutions were filter-sterilized independently before mixing the MOLP components. Semisolid MOLP agar was prepared the day before and air-dried (opened plates inside the biological hood) for at least 30 minutes prior to bacterial inoculation. *E*. *faecalis* MOLP-inoculated plates were incubated upside down in a highly humid environment to avoid dryness at 37°C for 6 days, unless indicated. After this period, semisolid media penetration was determined by removing all cells above the agar with 3 to 4 washes with ~10 mL distilled water and then observing bacterial growth within the agar.

When stated, MOLP media was solidified with poloxamer-407 (Sigma, Aldrich; MOLP-407), a fully autoclavable copolymer based on polyoxyethylene and polypropylene previously used for bacterial media growth development [84]. At low temperature, a poloxamer-407 solution is liquid, but becomes solid upon reaching room temperature (RT). MOLP-407 was prepared by the addition of 10 g of the polymer powder each day into 50 mL distilled water held at 4°C until a concentration of 60% [w/v] was achieved. This solution was then kept at 4°C for an extra 24 hours to ensure complete dissolution, prior to autoclaving. Next, it was cooled to RT and chilled to 4°C to liquefy. Once at low temperature, the poloxamer-407 solution was mixed 1:1 with a cold solution of 2X MOLP to a final volume of 100 mL. Subsequently, 1.5 mL of this chilled media were added into each well of 24-well plates (Falcon, Corning) and allowed to solidify at RT prior to inoculation with 1 μL of each bacterial strain grown overnight in TSB.

### Quantification of agar penetration

Colony forming units (CFU) of penetrating and non-penetrating *E*. *faecalis* cells grown on MOLP (for 6 days) solidified with either 1% (w/v) agar or 30% [w/v] poloxamer-407 were measured using two distinct strategies: To determine the number of external/internal cells grown on MOLP-407, the external cells from the colonies were collected and suspended in 500 μL of saline solution (0.89% NaCl). Subsequently, the surface of the plates was washed 3–4 times with 10 mL of sterile distilled water at RT, and invading bacteria were recovered by transferring the growth from each well into media previously chilled at 4°C to sterile Eppendorf tubes. All bacterial suspensions (internal and external cells) were centrifuged at 4°C and washed 3 times with ice-cold saline solution prior to making serial dilutions and plating on TSB agar plates. After 24 hours of incubation at 37°C, the final CFU number was calculated.

To quantify invasion of *E*. *faecalis* colonies grown on MOLP with 1% agar, the colonies were grown on top of 3.0 μm filters (Whatman) to separate the external from internal cells. The first ones (external) were collected by suspending each filter and suspended them in 500 μL of 1X Dulbecco’s Phosphate Buffered Saline solution (DPBS; Corning-Cellgro) and the remaining non-penetrating bacteria was removed by three washes with 10 mL of sterile distilled water and two washes with 70% ethanol (10 mL). The internal cells were recovered by removing an area of ~1 cm^2^ from the top layer, that was then suspended in 500 μL of DPBS as previously described above. Penetrating and non-penetrating bacterial suspensions were homogenized with a mortar and pestle followed a passage through a needle (27-gauge) syringe and filtered with a 40 μm nylon filter (BD Falcon). Final saline suspensions (1 mL) were sonicated for 2 minutes (30 seconds ON and OFF cycles) at 30% amplitude (Sonics Vibra Cell) to separate cellular clumps and then they were serially diluted and CFUs were determined as described above. Only when mutants exhibited growth differences to their parental strain, the final CFUs/mL was normalized to the absorbance (OD_600_) of each saline suspension from which serial dilutions were performed (Normalized CFUs/mL).

### Flow cytometry analyses

Internal and external cells of MOLP-grown colonies were recovered and processed as described above (see agar-penetration quantification section). Each penetrating and non-penetrating population was then diluted down to 0.5 OD_600_ prior to be stained with Brilliant Violet-570 (BV-570; LIVE/DEAD staining kit—Life technologies) for 30 min at RT in the dark, following the manufacturer’s instructions. Samples were washed twice with 1 mL DPBS and subsequently fixed with 4% paraformaldehyde (BioWorld) overnight at 4°C. Heat killed (100°C for 24 hours) TSB-grown *E*. *faecalis* was used as dead control. Live and dead bacteria were analyzed using a BD LSRII Flow cytometer.

### Scanning electron microscopy (SEM)

*E*. *faecalis* colonies were grown on MOLP as described above. SEM samples were prepared as previously described [85], with some minor modifications: External cells grown on MOLP were carefully transferred to ∼10 mm diameter pieces of 0.1% poly-L-lysine (Sigma, Aldrich) pre-treated Silicon wafers (Ted Pella). Samples were then fixed in a solution with 2.5% glutaraldehyde, 0.1% DMSO (dimethyl sulfoxide), 0.15% alcian blue and 0.15% safranin O [86], at RT for 18 hours. When stated, a 90 min post-fixation step with 1% Osmium tetroxide was performed. After three 15 min washes with distilled water and dehydration through a graded series of ethanol, the samples, unless specified were infiltrated with hexamethyldisilazane (HMDS; Sigma, Aldrich), through one incubation in 50% HMDS (in 100% ethanol) at RT for 1 hour and then two in 100% HMDS for 30 min. At this point the PDMS-bound samples were mounted on pins, dried under vacuum overnight, sputter-coated with gold–palladium alloy, and examined by SEM. For analyzing invading bacteria, small agar sections were placed on the silicon chips after removing the external cells with water; and treated as described above.

In order to objectively quantify the fraction of cells covered in matrix, the SEM images were analyzed using automatic image analysis software, Ilastik 1.3.0 [87]. The software was first trained to recognize different image structures, including background, matrix and cells, based on a single SEM image only. In this training stage, we manually identified regions in the image corresponding to background, matrix and cells, which the software uses to update a machine learning algorithm. After training, the algorithm was used to automatically analyze all remaining SEM images. In those images, pixels corresponding to either matrix or cells are automatically detected, thereby providing an estimate for fraction of cell surface that is covered in matrix. In all cases, we analyzed the SEM images at the same 20,000 X magnification. This magnification was chosen such that we could examine as much surface as possible, without compromising on the resolution needed for automatic image analysis.

### Genetic screening of mutants

A Mariner transposon insertion library in the multidrug resistant clinical isolate MMH594 [88] was constructed. *E*. *faecalis* was transformed with mariner delivery system pLB02 (kind gift of Dr. Lynn E. Hancock), which is identical to progenitor pCAM45 [89], except that erythromycin and kanamycin resistance markers were swapped for tetracycline and chloramphenicol resistance, respectively. Essentially as described in previous studies [89],^,^ transformants were initially selected at 30°C and the cure of the delivery vector was done at elevated temperature with selection for only the chloramphenicol resistance harbored by the transposon. A total of >300,000 MMH594 colonies possessing mariner insertions were collected as a pool.

To find targets necessary for enterococcal semisolid surface penetration, a replica-plating method was used. Approximately 6,000 mariner transposon mutants grown on TSB in 96-well plates for 24 hours were replica plated onto MOLP plates to screen for agar penetration capacity. A non-penetrating phenotype was designated as the ability to form WT-like colonies without growth inside the semisolid surface. To determine the genome site insertion of the mariner transposon, we used a previously described modified arbitrary PCR method with few modifications [90]. Amplification of short DNA-fragments was performed by using Platinum PCR High Fidelity SuperMix (Thermo Fisher Scientific) as described by the manufacturer. External and internal oligonucleotides specific for the Tn-Mariner (TnMextF1,and TnMxtF2; S4 Table) and the arbitrary primers (STAPHarb1, STAPHarb2, and STAPHarb3)[90] were utilized for the PCR reactions. The first round was performed using the arbitrary primers STAPHarb1 and SATPHarb2 (0.6 μM) paired with TnMexF1 (0.3 μM). 5.0 μL of a lysate of each mutant colonies obtained as previously described [91] was used for the PCR reaction: 95°C for 3 minutes; five cycles of 94°C for 30 seconds, 30°C for 30 seconds and 72°C for 1 min; then 25 cycles of 94°C for 30 seconds, 52°C for 30 seconds and 72°C for 1 minute; and finally 72°C for 5 minutes. Samples were kept at 4°C. The second PCR round was performed with primers TnMextF2 (0.3 μM) and STAPHarb3 (0.6 μM) as follow: 3 minutes at 94°C, 30 cycles of 94°C for 30 seconds, 55°C for 30 seconds and 72°C for 1 minute, followed by 72°C for 5 minutes. The samples were then kept at 4°C. The PCR products were visualized by agarose gel electrophoresis, and the second round PCRs containing at least one distinct visible fragment were used for further characterization. Nucleotide sequence analysis was performed with TnMextF2 primer. To identify of the Tn-Mariner insertion sites, a basic local alignment search tool (BLAST) was used.

### Plasmid construction and genetic complementation

The mariner transposon mutants, *glnA*::TnM and *rpiA*::TnM, were complemented in-trans by inserting the corresponding WT gene in the pAT28 plasmid [82]. To this end, PCR-amplified gene products with their corresponding promoters were generated for *rpiA* and *glnRA* from purified MMH594 genomic DNA. For *glnRA*, we used primers JD15 and JD30 (for sequences see S4 Table) to amplify a fragment of 2248 bp, which included a region 388 bp upstream of *glnR*, as well as *glnR* and *glnA* open reading frames (ORF). The PCR product was digested with *Eco*RI and *Bam*HI (NEB) and ligated into pAT28 to generate the complementation vector pJR01. Likewise, *rpiA* amplification was done using primers HV172 and HV173 (S4 Table). The amplified product (1291 bp) was digested with *Bam*HI and *Xba*I and ligated into pAT28 to generate the complementation vector pAH01. Plasmids, pJR01 and pAH01, were electroporated in *E*. *coli* and after sequencing several colonies; one was selected for complementation of each transposon mutant. The complementation vectors were transformed by electroporation into the corresponding *E*. *faecalis* strains and recovered on TSB plates (750 μg/mL spectinomycin) as previously described [78].

The fluorescence reporter strains were constructed by conjugation of the vector pV158-GFP between the donor *E*. *faecalis* OG1RF and the recipient MMH594 *rpiA*::TnM and *glnA*::TnM strains, as previously reported [92]. Briefly, TSB-grown overnight cultures of donor (15 μg/mL tetracycline) and recipients (10 μg/mL chloramphenicol) were diluted down to 0.05 OD_600_ and allowed to reach and absorbance of O.5 OD_600_. Then, the donor and recipient were mixed 1:10, 10:1 and 1:1 prior to concentrating these solutions to a final volume of 50 μL. Each one was finally placed onto a 0.2 μm-pore-size polycarbonate membrane (13 mm; Nucleopore) previously placed on TSB agar plates. After 24 hours at 37°C, filters were removed from the plates and placed in 1 ml 1X PBS (Dulbecco’s phosphate buffer saline; Sigma, Aldrich). Cells suspensions were then diluted and 10^−3^, 10^−7^ and 10^−9^ dilutions were plated on TSB agar with 15 μg/mL tetracycline, 10 μg/mL chloramphenicol and 250 μg/mL gentamycin. After 24 hours of incubation at 37°C, GFP fluorescent colonies were selected by microscopic analysis. The vector pV158-GFP was electroporated into electrocompetent cells of *E*. *faecalis* WT and Δ*epaX* strains prepared as previously described [93]. Cells were allowed to recover for 2 hours in 1 mL of SGM17MC recovery medium [93] before being plated and selected on TSB agar as described above.

### Generation of deletion mutants

*E*. *faecalis* MMH594 was used for the generation of the EF2170 (V583 *epaX* homolog) deletion mutant by allelic exchange (*ef2170*::*spcR*) using the pMINIMAD thermosensitive plasmid [81]. Briefly, fragments upstream and downstream of the EF2170 gene were PCR amplified with Phusion polymerase (NEB) using JD1, JD3; and JD6, JD8 primers, respectively. The spectinomycin resistance gene *spcR* was amplified from vector pIC333 [79] with primers JD4 and JD5. These three purified PCR fragments were assembled with Gibson Assembly Mix 2X (NEB) following the protocol suggested by the manufacturer. The final reaction was then used as template for amplifying a ~3 kb product (using primers JD2 and JD7) that was inserted into the *Bam*HI site of pMINIMAD generating the vector pJR02. This plasmid was then modified by inserting in the *Sal*I site, the chloramphenicol resistance gene *cat*, amplified from pLT06 [10] with primers JD44 and JD45 (S4 Table) generating pJR03. This last vector was then transformed into *E*. *coli* Top10 and transformants were selected after growth overnight in LB broth with 10 μg/mL chloramphenicol at 37°C. The plasmid pJR03 was purified and electroporated into WT MMH594 as previously described [78]. Transformed bacteria were grown on Todd-Hewitt agar plates with 15 μg/mL chloramphenicol at RT. Next, positive colonies were grown overnight on TSB with chloramphenicol at RT. Cells were centrifuged, suspended in 200 μl of fresh media, and plated in TSB agar with 400 μg/mL of spectinomycin. After 48 hours at 42°C, candidate colonies were grown on TSB agar with either 400 μg/mL of spectinomycin or 15 μg/mL chloramphenicol at RT. Allelic exchange was confirmed by PCR for the spectinomycin resistant and chloramphenicol sensitive colonies.

### Polysaccharide purification and analyses

Bacterial strains were cultured in 500 mL of either MOLP broth under static conditions to an OD_600_ of 0.6 or MOLP agar for 6 days at 37°C. Polysaccharides were extracted as previously described [46, 94] with minor modifications. Briefly, cells from either liquid cultures (for glycosyl composition analysis) or colonies were centrifuged 20 minutes at 4000 rpm and washed with 10 mL of sucrose-buffer (25% sucrose, 10 mM Tris-HCl; pH 8). Pellets were then suspended in 15 mL sucrose-buffer supplemented with 1 mg/mL lysozyme (Thermo Scientific) and 10 U/mL mutanolysin and incubated at 37°C overnight with gentle agitation. Following this incubation, the cellular fraction was removed by centrifugation for 20 minutes at 4500 rpm. The supernatants were treated with 200 μg/mL RNase A, 200 μg/mL DNase, 5 mM MgCl_2_, and 1 mM CaCl_2_ at 37°C for 8h to remove nucleic acids. Protein impurities were removed by adding proteinase K (50 μg/mL) to each supernatant and incubating them at 42°C overnight. Remaining contaminants were extracted using 1 mL of chloroform. The aqueous phase was transferred to a new tube following centrifugation (4500 rpm) for 15 minutes. Polysaccharides were precipitated by adding ethanol to a final concentration of 75% and incubation at − 80°C for 30 minutes, followed by a centrifugation (4500 rpm for 1 hour) at 4°C. Precipitated pellets were washed using 75% ethanol and allowed to air dry. Cell wall polysaccharide samples were submitted for glycosyl composition analysis to the Complex Carbohydrate Research Center (University of Georgia). Glycosyl composition analyses were performed using GC-MS of the per-O-trimethylsilyl (TMS) derivatives of the monosaccharide methyl glycosides. The TMS derivatives were produced from the sample by acidic methanolysis [95]. GC-MS analysis of TMS methyl glycosides was done on an Agilent 7890A GC interfaced to a 5975C MSD, using an Supelco Equity-1 fused silica capillary column (30 m x 0.25 mm ID). A total of 3 independent biological samples per strain were analyzed.

To visually analyze the composition of polysaccharides extracted from non-penetrating cells of *E*. *faecalis* VE14089 WT, Δ*epaX* and Δ*epaX* p-*epaX*, dry samples were suspended in 100 mL of Tris–NaCl (50 mM Tris-HCl, 150 mM NaCl; pH 8.0) and mixed with 16% glycerol prior to be run (25 μL) on a 10% polyacrylamide gel (acrylamide to bisacrylamide, 29:1; Fisher Scientific) in Tris-borate-EDTA buffer (89 mM Tris base, 89 mM boric acid, 2 mM EDTA; pH 8.0) for 90 minutes at 130 volts. Detection of polysaccharides was made with silver staining as previously described [46] with minor modifications. Briefly, the polyacrylamide gel was washed once with distilled water and incubated 45 minutes with 1 mg/mL of alcian blue in 3% acetic acid. Later, after three washes with distilled water, the gel was incubated in a solution with 3.4 mM K_2_Cr_2_O_7_ and 3.2 mM HNO_3_ for 7 minutes and then washed with water as described above. Following these steps, the gel was then treated with 12mM AgNO_3_ and exposed to intense light for 30 min. It was later washed with water, soaked in 50 mL of 0.28 M Na_2_CO_3_ and 6mM formaldehyde until signal was visually detected and transferred to a solution of 0.1 M acetic acid for storage.

### Immunochemical detection of polyGlcNAc-containing polysaccharides

Bacterial samples from either external or penetrating *E*. *faecalis* grown on MOLP for 6 days were collected and processed as previously described [8], with the following modification: Cells were suspended in 1000 μL of DPBS and then spotted onto microscope slides. After samples air-dried, they were fixed by methanol: acetone 1:1 for 10 min at -20°C. Then, they were treated with 100% ice-cold methanol for ~1 min, followed by 3 washes with PBS-NaCl buffer (20 mM PBS and 150 mM NaCl). Samples were blocked with 2.5% Normal Horse serum (Vectors Lab) for 45 min at RT. PBS-1% BSA (bovine serum albumin; Sigma, Aldrich) was then added after removing the blocking serum. After 1 min incubation at RT, slides were reacted overnight at 4°C with 20 μg/mL of human IgG mAb F598, which specifically binds to β-1,6-linked GlcNAc polysaccharides [8, 52]. After 3 washes with PBS-NaCl, samples were reacted with the secondary antibody, anti-human IgG labeled with Alexa Fluor-488 (15 μg/mL; Invitrogen) and DAPI (2.0 μg/mL), for 2 hours at RT. To visualize GlcNAc residues, samples were incubated with 5 μg/mL of the lectin WGA (wheat germ agglutinin) directly conjugated to Texas Red (Thermo Fisher Scientific) for 30 min at RT. Slides were then washed and cover-slipped using Fluoromount-G media (SouthernBiotech). Images were captured at 63x magnification at 1000 ms for DAPI, and 600–1500 ms for FITC and Rhodamine. Imaging was performed with a Zeiss AxioObserver inverted wide field/fluorescence microscope and processed using MetaMorph software. All images were adjusted to reduce background fluorescence.

For enzymatic treatments, cell samples obtained as described above were diluted 1:10 in PBS. 10 μL of these cell solutions were suspended in 90 μL of Tris-buffered saline (TBS; pH 7.4) containing 300 μg/mL DspB [96]. Samples were incubated for 24 hours at 37°C with constant shaking, and then centrifuged to suspend the pelleted cells into 50 μL of fresh TBS. Each suspension was subsequently placed onto glass slides to then be treated as described above.

### Colony immunoblotting assay

*E*. *faecalis* colonies were analyzed for extracellular production of polyGlcNAc-polymers using a protocol previously described [97] with some modifications: Briefly, 0.45 μm nitrocellulose membranes (Bio-Rad) were placed on 1-day-old colonies grown on MOLP until they became completely wet. The plates/membranes were incubated at 37°C for 10 minutes prior to be carefully removed and transfer colony side up to a glass petri dish for air-drying (10 minutes at 37°C). Following this step, the air-dried membranes were immersed in chloroform at RT for ~15 minutes or until the chloroform completely evaporated. Each nitrocellulose membrane was incubated colony side down for 60 minutes in the blocking buffer (25mM Tris-base, 0.15M NaCl, 0.1% Tween-20, 5% non-fat milk). After 3 washes, 5 minutes each with TBS-T (25mM Tris-base, 0.15M NaCl, 0.1% Tween-20), the membranes were incubated overnight at 4°C with gentle agitation in TBS-T with 5% bovine serum albumin (BSA) and 200 μg/mL of the primary antibody mAb F598. Membranes were washed with TBS-T as described above and then incubated for 60 minutes in TBS-T containing a 1/10000 dilution of peroxidase-conjugated goat anti-human IgG polyclonal antiserum (Thermo Fisher Scientific). Membranes were then washed 3 times for 5 min each with TSB-T and were developed using the SuperSignal West Pico Chemioluminescent Substrate Kit as directed by manufacturer (Thermo Fisher Scientific).

### Nanostring analysis of *E*. *faecalis* glycosyltransferases

External and penetrating cells, from 2-day-old colonies grown on MOLP were collected and suspended in 2 mL of cold RNAlater. Samples were pelleted and supernatant was discharged prior to storage at -80°C. For cell lysis, these pellets were suspended in RLT buffer (500 μL; Qiagen) and completely disrupted with a beat beater in one volume 0.5 mm zilica/sirconia beads for ~4 minutes (4 times × 60 seconds). Cellular debris were removed and supernatants were then subjected to probe hybridization and processing with the Nanostring nCounter Prep Station and Digital Analyzer according to the manufacturer’s instructions. Raw code counts were analyzed according to manufacturer’s guidelines; briefly, total transcript counts were normalized using internal controls with background subtraction. Transcript counts for 5 genes (*gyrB*, *def*, *sigA*, *aqpZ* and *folB*) were used for geometric mean normalization to correct for differences in total mRNA concentration. All data were collected from 2 biological replicates and gene expression was considered significantly altered if the transcript number changed >2-fold. Total counts were expressed as log_2_-change relative to the counts of non-penetrating cells at day 1, a time point where invasion was not evidenced in MOLP.

### Translocation assays

T84 human intestinal epithelial cells (Sigma, Aldrich) were grown and maintained as previously described [49] with some modifications. Briefly, cell monolayers were grown on plastic in a 1:1 Dubelcco’s Modified Eagle’s medium and nutrient mixture F-12 (DMEM/F12; Corning Inc.) supplemented with 10% heat inactivated fetal bovine serum (FBS; Atlanta biologicals), 2 mM glutamine, 1 mM sodium piruvate, 10 mM HEPES buffer (pH 7), 1X non-essential amino acids, 50 units/mL penicillin and 50 μg/mL streptomycin (Corning Inc.), 5 μg/mL prophylactic plasmocin (InvivoGen), and 0.007% β-mecaptoethanol (Sigma, Aldrich). When monolayers reached confluence or near-confluence, cells were detached and split as previously described [98]. Translocation was performed by seeding 10^5^ T84 human epithelial cells from previous passages into a 24-well Transwell system with 3.0-μm-pore-size polycarbonate membranes (Corning Costar Corp). This pore size allows bacteria, but not T84 cells, to penetrate the membrane. A volume of 300 and 1000 μL of the tissue culture medium described above was added to the apical and basolateral chambers, respectively; and this medium was changed every 2–3 days. The developing progress of T84 tight junctions was monitored by Millicell-ERS-2 measurement (Millipore). Translocation experiments were performed after 8 days of culture, when the trans-epithelial electrical resistance (TER) of T84 monolayers reached ~8000 Ω/cm2 or higher. To prepare bacteria for translocation, 12-hours-bacterial cultures (with appropriate media and antibiotics) were diluted down in HBSS (Hanks balanced salt solution without Ca^2+^ and Mg^2+^; Corning Inc.) to an absorbance of 0.25 OD_600_ (~10^8^ CFU/mL). Bacterial solutions were then washed twice with 1 mL of HBSS and finally suspended in Translocation Media (TM; Gibco): Advanced DMEM/F-12 mixture supplemented with 5% FBS, 10 mM HEPES buffer (pH 7), 0.007% β-mecaptoethanol and when specified, 2 mM GluN. Prior to bacterial inoculation, the filters were washed twice with TM. After this step, 1000 μL of fresh medium were added to the basolateral chamber, and 300 μL of each TM-suspended bacterial culture prepared as described above, were inoculated to the apical side of the chamber; this inoculum is consistent with that used by others [49] and with the density of intestinal enterococci in some settings. TER was monitored at the beginning and after 8 hours post-infection. The TER values remained similar to those obtained for the pre-infected monolayers, indicating that the integrity of cell barriers was conserved throughout the experiments. CFUs of viable bacteria in both chambers were counted at 0, and 8 hours by removing 20 μl aliquots, serially diluting and plating on TSB agar plates. For each strain, 8–9 independent transwells were used and the experiments were repeated at least three times.

To visualize translocating bacteria, filters seeded with polarized human enterocyte-like T84 cells as described above were infected for 2-hours with *E*. *faecalis* constitutively expressing GFP, and then samples (infected and uninfected) were stained and observed by laser scanning confocal microscopy. Bacteria and epithelial cell translocation assays were done in TM supplemented with 15 μg/mL tetracycline. For immunofluorescence staining, medium on each transwell was removed and filters were washed two times with pre-warmed (37°C) PBS. Cells were then fixed by 4% paraformaldehyde for 40 min. Following fixation, cells were washed with DPBS for 30 seconds, and permeabilized by incubating them with PBT (PBS and 0.5% TritonX-100) solution for 15 minutes. The solution was removed and the cells (transwells) were washed twice with DPBS for 30 seconds. After this, samples were blocked with PBS-1% BSA for 1 hour at RT, and then washed once with PBS as previously described. Cells were reacted overnight at 4°C with 20 μg/mL of MAb F598 [8, 52]. After three washes with PBS (5 minutes) samples were incubated with the secondary antibody, goat anti-human IgG labeled with Alexa Fluor-647 (15 μg/mL; Invitrogen) for 2 hours at RT. Thereafter, cells were washed three times with PBS (5 minutes), followed by incubation with cellular dyes (200nM of both Alexa Fluor 594-coupled phalloidin and DAPI; Invitrogen) in PBS containing for 30 min in the dark at RT. The solution was removed and samples were washed three times with PBS for 30 seconds. Filters were cut and transferred into ~10 μL of ProLong Diamond Antifade Mountant (Thermo Fisher), followed by sealing on glass slides and storing in the dark at 4°C until microscopy. Imaging was performed with a LSM880 confocal microscope and processed using Image J software. All images were adjusted to reduce background fluorescence.

### Statistical analyses

Unless noted otherwise, all experiments were repeated at least three times and results were similar between repeats. All statistical analyses were determined using GraphPad Prism 7.0. Differences between the means of experimental groups were calculated using either a two-tailed unpaired Student’s t-test or one-way analysis of variance (ANOVA). Error bars represent SEM from independent samples assayed within the represented experiments. *P*<0.05 was considered to be statistically significant.

## Supporting information

S1 Fig *Enterococcus faecalis* penetrates different semisolid surfaces in a time-dependent manner. Strain MMH594 (unless specified otherwise) was grown on MOLP solidified with either 1% agar (**A**, **B** and **E**), agarose (**C**) or 30% poloxamer-407 (MOLP-407; **D**) for 6 days at 37°C. Enterococcal penetration was evidenced as a colony-print inside the agar after removing the external cells. (**A**) Colonies (*outside*) and bacterial penetration areas (*inside*) of *E*. *faecalis* clinical isolates V583, JH2-2, 12030, MMH594 and VE14089, and the human commensal OG1RF. (**B**) Time-lapse analysis of *E*. *faecalis* generating colony-prints over a period of 144 hours (6 days). Scale bars **A** and **B**: 6,000 μm. (**C**) External or penetrating *E*. *faecalis* cells (on MOLP with agarose concentrations ranging from 0.2 to 1.0%. Lower scale bar: 5,000 μm. (**D**) External (*outside*) and internal (*inside*) *E*. *faecalis* cells grown on MOLP-407 (*top*) were determined by plating bacterial serial dilutions on TSB agar plates and by quantifying colony forming units (CFUs/mL) after 24 hours of growth (*bottom*; mean±SE; n = 4; *P<0.05; two-tailed unpaired *t*-test). Scale bar: 2,000 μm. Transmitted light images of agar sections from the center (**E**) or edge (**F**) areas of the penetrating colony-print. Top white bar indicates the beginning of the agar in each section. Lower scale bar: 60 μm. Yellow lines indicate places where the depth of the microcolony was measured.
(PDF)

S2 Fig Growth and agar penetration by *E. faecalis* mutants. (**A**) OG1RF WT or pili-deficient mutants Δ*ebpABC* and Δ*ebpA* were grown on MOLP. Penetration was evidenced after washes with distilled water. Lower scale bar: 2,500 μm. (**B**) *E*. *faecalis* MMH594 WT, *glnA*::TnM and *rpiA*::TnM were grown in MOLP broth for 48 hours with constant agitation at 37°C. Enterococcal growth was determined by measuring the absorbance at 600 nm at different time points (mean±SE; n = 10). (**C**) Quantification of colony forming units (CFUs/mL) of non-penetrating cells of MOLP-grown colonies from WT, *glnA*::TnM or *rpiA*::TnM strains with or without the empty vector pAT28, or *in-trans* complemented mutants with pAT28 harboring their corresponding WT allele (p-*glnA* and p-*rpiA*) (mean±SE; n = 6; non-significant, ns, *P*>0.05; ****P<0.0001 for both the one-way ANOVA and Tukey’s multiple comparison test). The total CFUs/mL were normalized to the initial absorbance (OD_600_) prior to making serial dilutions.
(PDF)

S3 Fig Characterization of strains producing polyGlcNAc-containing polymers. (**A**) Immunofluorescence analysis of WT *S*. *aureus* MN8 (positive control) or Δ*ica* (PNAG (PIA)-deficient strain) grown for 24 hours on Columbia-blood medium incubated with mAb F598. To visualize antibody binding to polyGlcNAc-containing polymers, cells were reacted with anti-human IgG antibodies conjugated to Alexa Fluor-488 (green fluorescence). DAPI was used to stain bacterial DNA (blue fluorescence). To visualize other GlcNAc residues, cells were also treated WGA conjugated to Texas Red (red fluorescence). Scale bar: 20 μm. (**B**) Immunoblot of WT, *glnA*::TnM or *rpiA*::TnM colonies grown on semisolid MOLP with or without 10 mM GlcNAc. Scale bar: 1,000 μm. (**C**) The relative intensity obtained upon incubation with mAb F598 was calculated for each colony using Image J (mean±SE; n = 8; ****P<0.0001 for both the overall one-way ANOVA and Tukey’s multiple comparison test). (**D**) Fluorescence phenotypes of WT *E*. *faecalis* MMH594 (DK8) and VE14089 (DK1) colonies grown on MOLP with 0.02% calcofluor white (CFW), a fluorescent dye binding surface polysaccharides harboring β-1,3 and β-1,4 linkages. The fungus *Candida albicans* and the bacterium *Escherichia coli* grown on MOLP for 48 hours were used as positive and negative controls, respectively. All CFW plate growth and incubation experiments were performed in the dark, and CFW reactivity was visualized by long-wave UV light (*lower panel*).
(PDF)

S4 Fig EpaX is necessary for semisolid surface penetration. (**A**) Nanostring analysis of penetrating (*inside*) and non-penetrating (*outside*) cells from *E*. *faecalis* MMH594 grown for 2 days on MOLP at 37°C. Gene annotations or names based on *E*. *faecalis* V583 genome database are shown on the left of the heat map, including 13 putative glycosyltransferases and the acetyltransferase EF0590. Normalized mRNA counts are expressed compared with their expression in non-invading one-day-old cells grown on MOLP. Color legend for Log_2_ expression is shown below. (**B**) *E*. *faecalis* VE14089 WT and Δ*epaX* were grown in MOLP broth for 48 hours with constant shaking at 37°C. Enterococcal growth was determined by measuring the absorbance at 600 nm at different time points (mean±SE; n = 10). (**C**) Images of colonies outside or penetrating cells of strains grown for 6 days at 37°C. Penetration was tested for ΔEF2170 (Δ*epaX*) and its parental strain *E*. *faecalis* MMH594 in the presence or absence exogenous 10 mM GlcNAc. Scale bar: 6,000 μm. (**D**) Polysaccharide characterization of WT VE14089, Δ*epaX*, and its genetically complemented strain (Δ*epaX* p-*epaX*). Oligosaccharides were extracted from six-day-old colonies grown on semisolid MOLP and visualized in a 10% polyacrylamide gel stained with alcian blue and silver nitrate staining. The head arrows indicate the bands corresponding to the different polysaccharides detected for each strain analyzed. (**E**) Immunofluorescence analysis of *E*. *faecalis* WT and Δ*epaX* cells from MOLP-grown colonies treated with mAb F598. (green fluorescence). DAPI was used to stain bacterial DNA (blue fluorescence). To visualize GlcNAc and sialic acid residues cells were also treated with WGA (red fluorescence). Scale bar: 20 μm. (**F** and **G**) 1 μL of an TSB-grown overnight culture of *E*. *faecalis* VE14089 and Δ*epaX* was inoculated on MOLP with and without 200 μM PNAG purified from *S*. *aureus* MN8. Colonies were imaged after 6 days of growth. Scale bar: 5,000 μm (**F**; *left*). Quantification of cells above the agar was determined and expressed as CFUs/mL (**G**; *right*; mean±SE; n = 3).
(PDF)

S5 Fig PolyGlcNAc-containing polysaccharides are necessary for efficient *E. faecalis* translocation through T84 human epithelial cell monolayers. (**A** and **B**) Colony forming units (CFUs/mL) of viable cells that did not pass through the monolayer (apical side) or translocated to the basolateral side after 8 hours of incubation. *E*. *coli* DH5α was used as a negative control (mean±SE; n = 5; ****P<0.0001 for both the one-way ANOVA and Turkey’s multiple comparison test). (**C** and **D**) 3D reconstruction (x, y, z) of confocal immunofluorescence images of T84 cell monolayers uninfected (*left*) or infected for 2 hours with either WT *E*. *faecalis* MMH594 (*center*), *rpiA*::TnM (**C**) or *glnA*::TnM mutants (**D**; *right*) constitutively expressing GFP (green fluorescence). Alexa Fluor 594-coupled phalloidin and DAPI were used to stain the epithelial cell actin (red fluorescence) and nucleus (blue fluorescence), respectively. To visualize polyGlcNAc-containing polymers, T84 epithelial cells and enterococci co-cultures were treated with mAb F598 and subsequently reacted with anti-human IgG conjugated to Alexa Fluor-647 (gray fluorescence; white arrow). The yellow lines mark the intersection point were the x, z (*top*) and y, z (left) orthogonal views of reconstructed Z-sections were taken. Scale bar: 5 μm. The orange square represents a 2X-magnified view of the selected area in x, y planes (green square). *E*. *faecalis* translocation assays and microscopy assays were done in media with (**A** and **C**) or without exogenous glutamine (**B** and **D**).
(PDF)

S6 Fig Mutations in EpaB do not affect *E. faecalis* agar penetration or translocation through epithelial cell monolayers. (**A**) Colony forming units (CFUs/mL) of viable cells in the apical side or translocated to the basolateral side after 8 hours of incubation. *E*. *coli* DH5α was used as negative control. (mean±SE; n = 8; ns, *P* >0.05; *t*-test). (**B**) Immunofluorescence analysis of six-day-old WT *E*. *faecalis* and Δ*epaB* colonies incubated with the mAb F598 antibody. To visualize antibody binding to polyGlcNAc-containing polymers, cells were reacted with the anti-human IgG antibodies conjugated to Alexa Fluor-488 (green fluorescence). DAPI was used to stain bacterial DNA (blue fluorescence). To visualize GlcNAc residues cells were also treated WGA conjugated to Texas Red (red fluorescence) Scale bar: 20 μm. (**C**) Colony immunoblot (*top panel*) of *E*. *faecalis* mutant and their parental strain grown on MOLP for 24 hours. *S*. *aureus* MN8 was used as positive control. The relative intensity obtained upon hybridization with mAb F598 was calculated for each colony using Image J (*lower panel*); (mean±SE; n = 8; ns, *P*>0.05 for both the overall one-way ANOVA and Tukey’s multiple comparison test). Scale bar: 1,000 μm. (**D**) Analysis of MOLP penetration (inside) by *E*. *faecalis* OG1RF WT and mutant Δ*epaB*.
(PDF)

S1 Table One-way ANOVA and Turkey’s multiple comparison test of the non-invading cells. Mean±SE; n = 6; *P<0.05; **P<0.01; ***P<0.001; **** P<0.0001 for both the one-way ANOVA and Tukey’s multiple comparison test; nonsignificant, ns.
(PDF)

S2 Table List of putative genes evaluated by Nanostring analysis. (PDF)

S3 Table Strains and plasmid list used in this study. Amp^r^, ampicillin resistant, Rif^r^, rifampin resistant; Fus^r^, fusidic acid resistant; Sp^r^, spectinomycin resistant; Tet^r^, tetracycline resistant; Cm^r,^ chloramphenicol resistant; Em^r^, erythromycin resistant. *E*. *faecalis* MMH594 strains harboring pAT28 vector and derivatives required 750 μg/mL spectinomycin. Transposon insertion strains required 10 μg/mL chloramphenicol. Fluorescent reporter strains harboring pMV158 derivatives needed 15 μg/mL tetracycline. *E*. *faecalis* harboring pLT06 derivatives required 15 μg/mL chloramphenicol. pMINIMAD derivatives required 100 μg/mL ampicillin. WT: Wild type.
(PDF)

S4 Table List of primers used in this study. Restriction sites are underlined
(PDF)
