# Supplementary figures and images for: PolyGlcNAc-containing exopolymers enable surface penetration by non-motile Enterococcus faecalis
Source: PLoS Pathog. 2019 Feb 11;15(2):e1007571. doi: 10.1371/journal.ppat.1007571 (PMC6386517; doi:10.1371/journal.ppat.1007571)

**S1 Fig.**

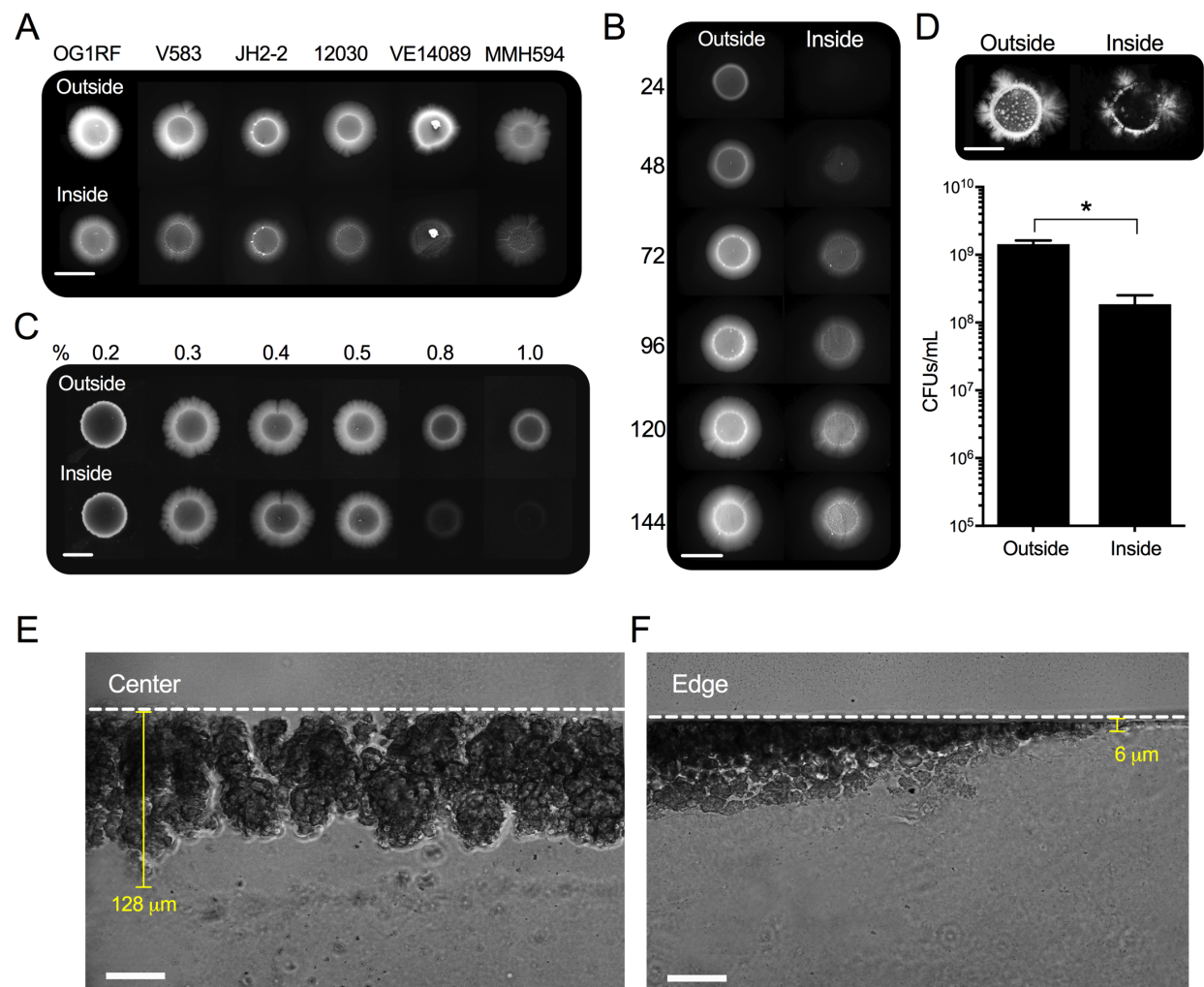

Supplement: S1 Fig — Strain MMH594 (unless specified otherwise) was grown on MOLP solidified with either 1% agar (A, B and E), agarose (C) or 30% poloxamer-407 (MOLP-407; D) for 6 days at 37°C. Enterococcal penetration was evidenced as a colony-print inside the agar after removing the external cells. (A) Colonies (outside) and bacterial penetration areas (inside) of E. faecalis clinical isolates V583, JH2-2, 12030, MMH594 and VE14089, and the human commensal OG1RF. (B) Time-lapse analysis of E. faecalis generating colony-prints over a period of 144 hours (6 days). Scale bars A and B: 6,000 μm. (C) External or penetrating E. faecalis cells (on MOLP with agarose concentrations ranging from 0.2 to 1.0%. Lower scale bar: 5,000 μm. (D) External (outside) and internal (inside) E. faecalis cells grown on MOLP-407 (top) were determined by plating bacterial serial dilutions on TSB agar plates and by quantifying colony forming units (CFUs/mL) after 24 hours of growth (bottom; mean±SE; n = 4; *P<0.05; two-tailed unpaired t-test). Scale bar: 2,000 μm. Transmitted light images of agar sections from the center (E) or edge (F) areas of the penetrating colony-print. Top white bar indicates the beginning of the agar in each section. Lower scale bar: 60 μm. Yellow lines indicate places where the depth of the microcolony was measured. (PDF) [file ppat.1007571.s001.pdf]

S2 Fig.

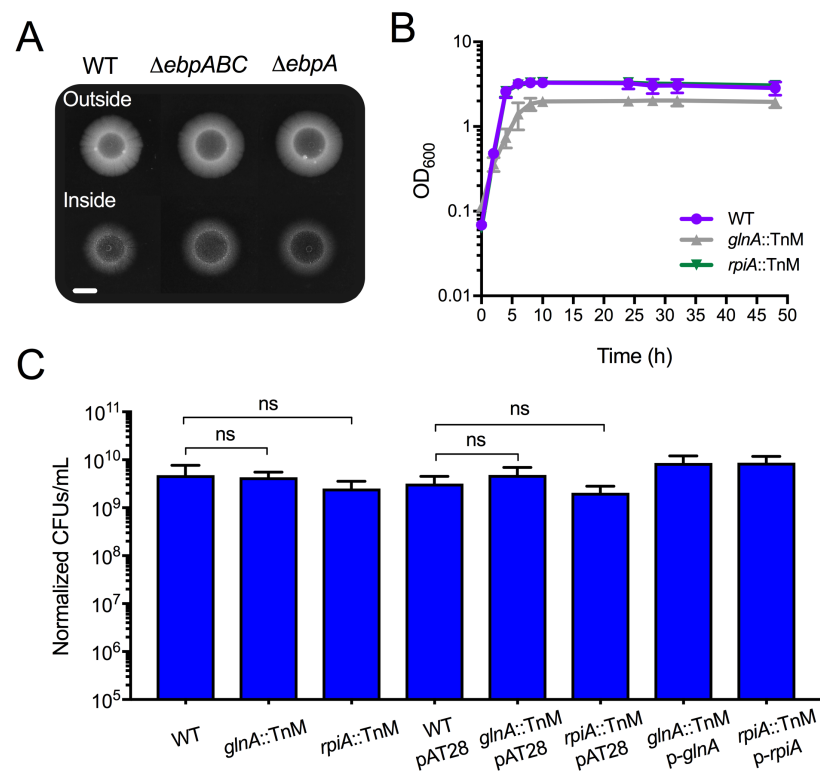

Supplement: S2 Fig — (A) OG1RF WT or pili-deficient mutants ΔebpABC and ΔebpA were grown on MOLP. Penetration was evidenced after washes with distilled water. Lower scale bar: 2,500 μm. (B) E. faecalis MMH594 WT, glnA::TnM and rpiA::TnM were grown in MOLP broth for 48 hours with constant agitation at 37°C. Enterococcal growth was determined by measuring the absorbance at 600 nm at different time points (mean±SE; n = 10). (C) Quantification of colony forming units (CFUs/mL) of non-penetrating cells of MOLP-grown colonies from WT, glnA::TnM or rpiA::TnM strains with or without the empty vector pAT28, or in-trans complemented mutants with pAT28 harboring their corresponding WT allele (p-glnA and p-rpiA) (mean±SE; n = 6; non-significant, ns, P>0.05; ****P<0.0001 for both the one-way ANOVA and Tukey’s multiple comparison test). The total CFUs/mL were normalized to the initial absorbance (OD600) prior to making serial dilutions. (PDF) [file ppat.1007571.s002.pdf]

S3 Fig.

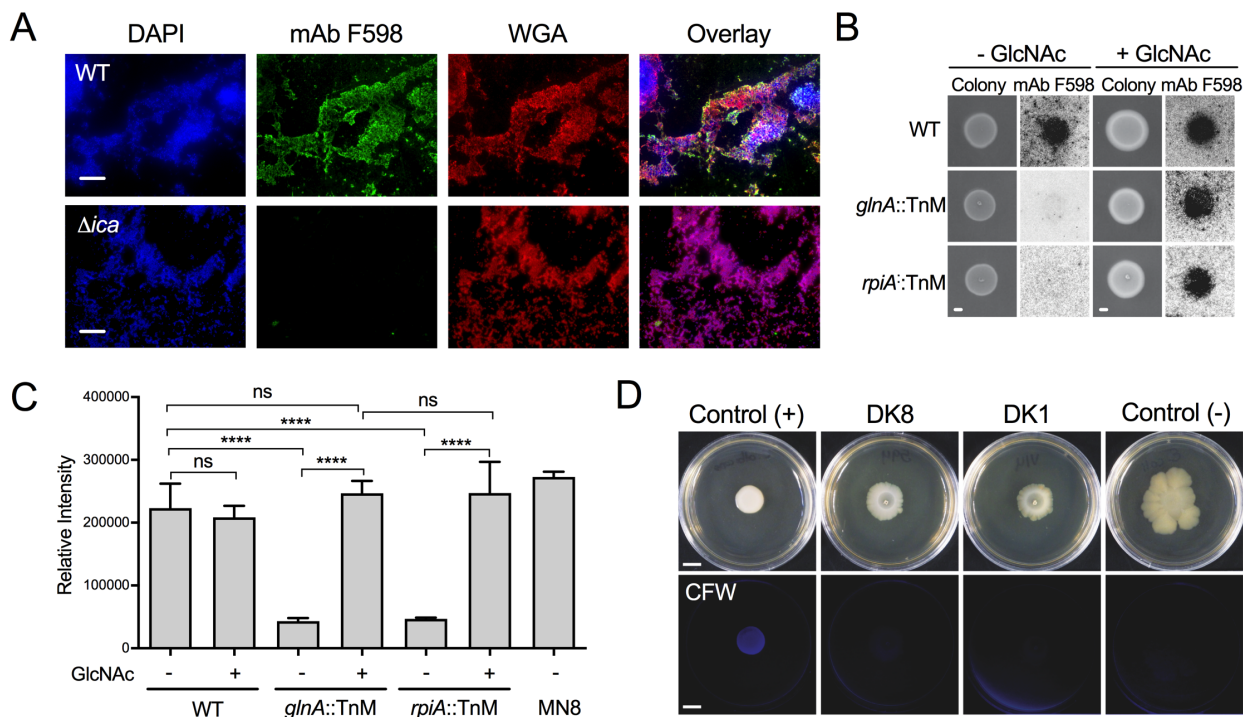

Supplement: S3 Fig — (A) Immunofluorescence analysis of WT S. aureus MN8 (positive control) or Δica (PNAG (PIA)-deficient strain) grown for 24 hours on Columbia-blood medium incubated with mAb F598. To visualize antibody binding to polyGlcNAc-containing polymers, cells were reacted with anti-human IgG antibodies conjugated to Alexa Fluor-488 (green fluorescence). DAPI was used to stain bacterial DNA (blue fluorescence). To visualize other GlcNAc residues, cells were also treated WGA conjugated to Texas Red (red fluorescence). Scale bar: 20 μm. (B) Immunoblot of WT, glnA::TnM or rpiA::TnM colonies grown on semisolid MOLP with or without 10 mM GlcNAc. Scale bar: 1,000 μm. (C) The relative intensity obtained upon incubation with mAb F598 was calculated for each colony using Image J (mean±SE; n = 8; ****P<0.0001 for both the overall one-way ANOVA and Tukey’s multiple comparison test). (D) Fluorescence phenotypes of WT E. faecalis MMH594 (DK8) and VE14089 (DK1) colonies grown on MOLP with 0.02% calcofluor white (CFW), a fluorescent dye binding surface polysaccharides harboring β-1,3 and β-1,4 linkages. The fungus Candida albicans and the bacterium Escherichia coli grown on MOLP for 48 hours were used as positive and negative controls, respectively. All CFW plate growth and incubation experiments were performed in the dark, and CFW reactivity was visualized by long-wave UV light (lower panel). (PDF) [file ppat.1007571.s003.pdf]

S4 Fig.

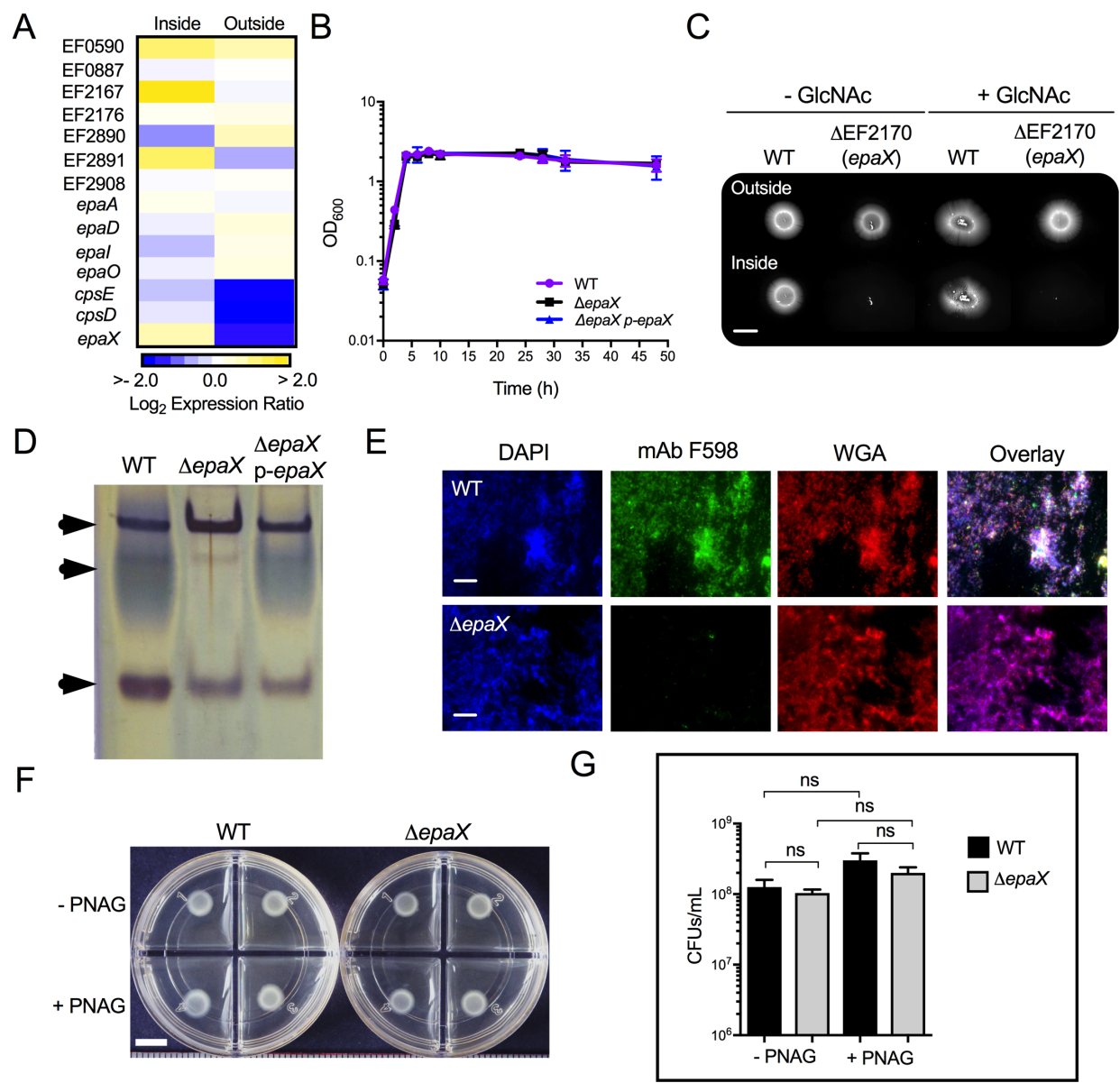

Supplement: S4 Fig — (A) Nanostring analysis of penetrating (inside) and non-penetrating (outside) cells from E. faecalis MMH594 grown for 2 days on MOLP at 37°C. Gene annotations or names based on E. faecalis V583 genome database are shown on the left of the heat map, including 13 putative glycosyltransferases and the acetyltransferase EF0590. Normalized mRNA counts are expressed compared with their expression in non-invading one-day-old cells grown on MOLP. Color legend for Log2 expression is shown below. (B) E. faecalis VE14089 WT and ΔepaX were grown in MOLP broth for 48 hours with constant shaking at 37°C. Enterococcal growth was determined by measuring the absorbance at 600 nm at different time points (mean±SE; n = 10). (C) Images of colonies outside or penetrating cells of strains grown for 6 days at 37°C. Penetration was tested for ΔEF2170 (ΔepaX) and its parental strain E. faecalis MMH594 in the presence or absence exogenous 10 mM GlcNAc. Scale bar: 6,000 μm. (D) Polysaccharide characterization of WT VE14089, ΔepaX, and its genetically complemented strain (ΔepaX p-epaX). Oligosaccharides were extracted from six-day-old colonies grown on semisolid MOLP and visualized in a 10% polyacrylamide gel stained with alcian blue and silver nitrate staining. The head arrows indicate the bands corresponding to the different polysaccharides detected for each strain analyzed. (E) Immunofluorescence analysis of E. faecalis WT and ΔepaX cells from MOLP-grown colonies treated with mAb F598. (green fluorescence). DAPI was used to stain bacterial DNA (blue fluorescence). To visualize GlcNAc and sialic acid residues cells were also treated with WGA (red fluorescence). Scale bar: 20 μm. (F and G) 1 μL of an TSB-grown overnight culture of E. faecalis VE14089 and ΔepaX was inoculated on MOLP with and without 200 μM PNAG purified from S. aureus MN8. Colonies were imaged after 6 days of growth. Scale bar: 5,000 μm (F; left). Quantification of cells above the agar was determined and expressed as CFUs/mL [file ppat.1007571.s004.pdf]

S5 Fig.

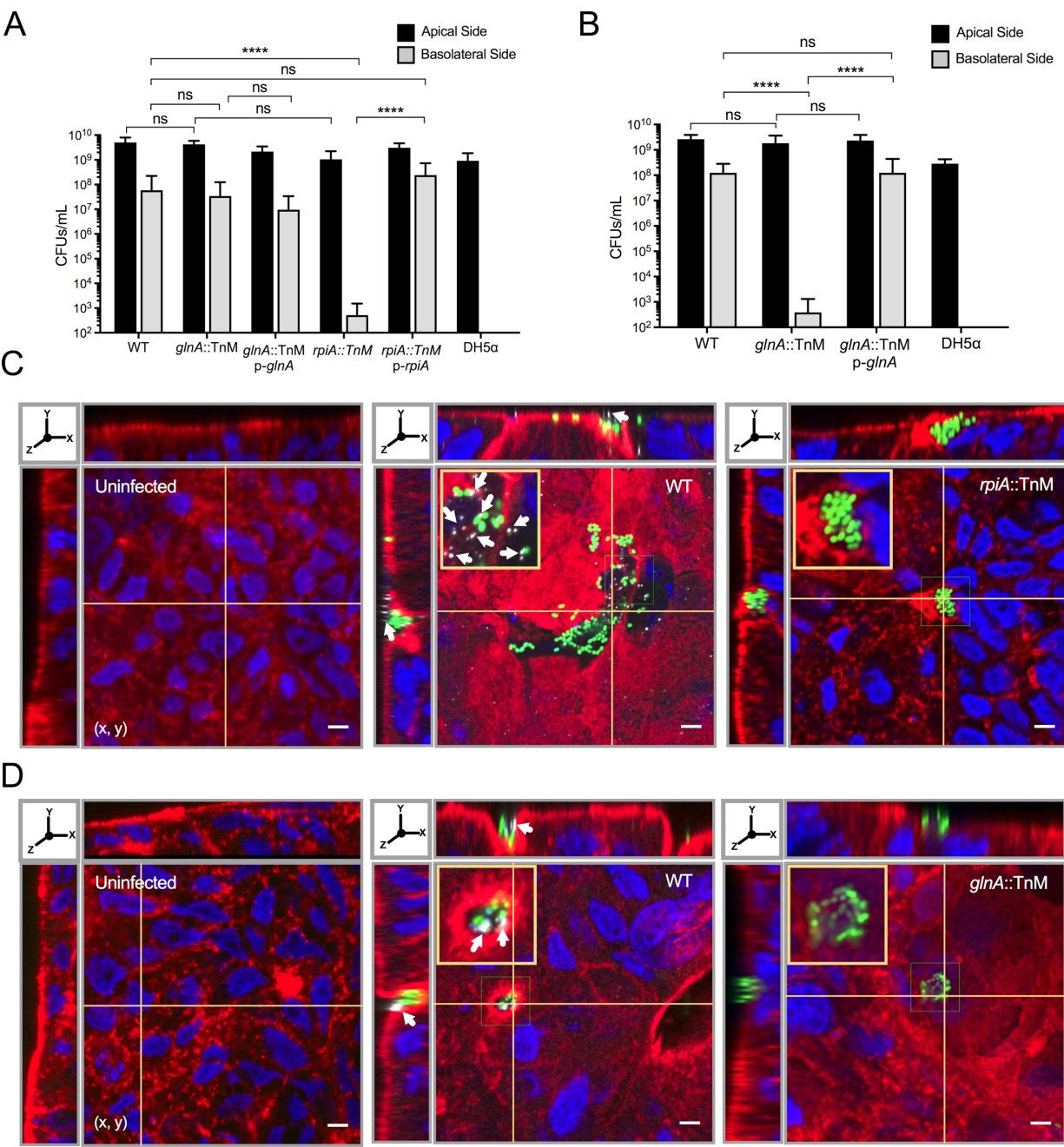

Supplement: S5 Fig — (A and B) Colony forming units (CFUs/mL) of viable cells that did not pass through the monolayer (apical side) or translocated to the basolateral side after 8 hours of incubation. E. coli DH5α was used as a negative control (mean±SE; n = 5; ****P<0.0001 for both the one-way ANOVA and Turkey’s multiple comparison test). (C and D) 3D reconstruction (x, y, z) of confocal immunofluorescence images of T84 cell monolayers uninfected (left) or infected for 2 hours with either WT E. faecalis MMH594 (center), rpiA::TnM (C) or glnA::TnM mutants (D; right) constitutively expressing GFP (green fluorescence). Alexa Fluor 594-coupled phalloidin and DAPI were used to stain the epithelial cell actin (red fluorescence) and nucleus (blue fluorescence), respectively. To visualize polyGlcNAc-containing polymers, T84 epithelial cells and enterococci co-cultures were treated with mAb F598 and subsequently reacted with anti-human IgG conjugated to Alexa Fluor-647 (gray fluorescence; white arrow). The yellow lines mark the intersection point were the x, z (top) and y, z (left) orthogonal views of reconstructed Z-sections were taken. Scale bar: 5 μm. The orange square represents a 2X-magnified view of the selected area in x, y planes (green square). E. faecalis translocation assays and microscopy assays were done in media with (A and C) or without exogenous glutamine (B and D). (PDF) [file ppat.1007571.s005.pdf]

**S6 Fig.**

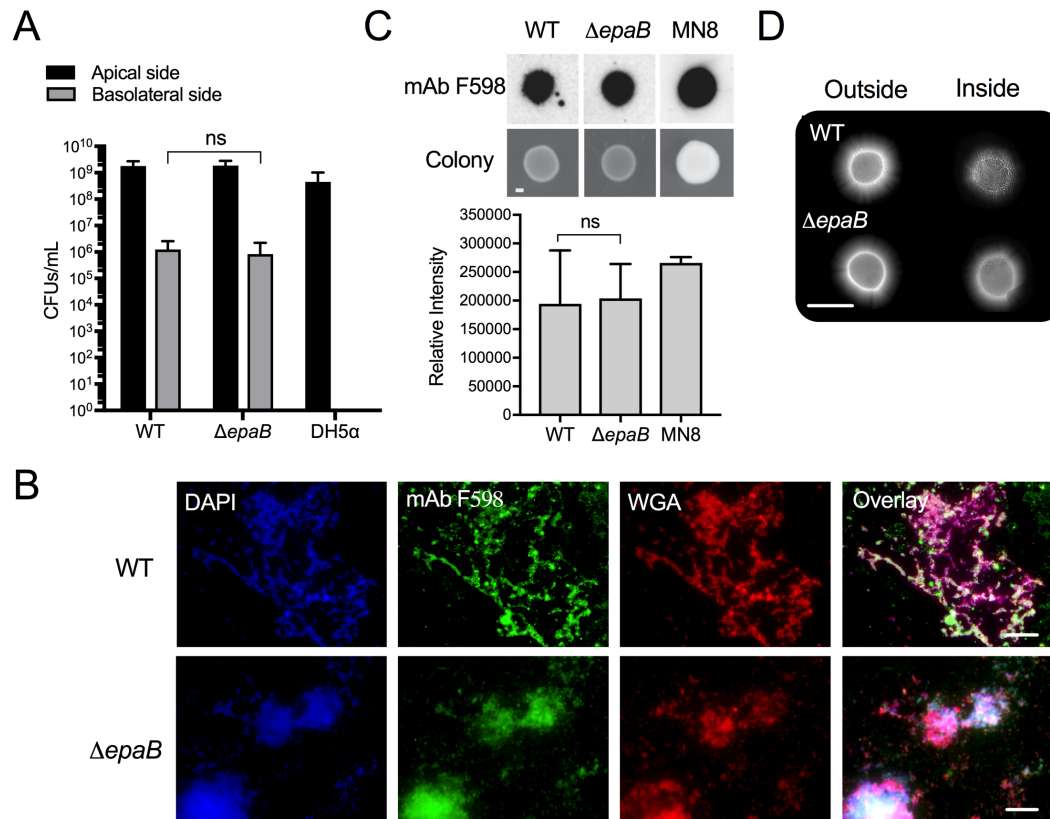

Supplement: S6 Fig — (A) Colony forming units (CFUs/mL) of viable cells in the apical side or translocated to the basolateral side after 8 hours of incubation. E. coli DH5α was used as negative control. (mean±SE; n = 8; ns, P >0.05; t-test). (B) Immunofluorescence analysis of six-day-old WT E. faecalis and ΔepaB colonies incubated with the mAb F598 antibody. To visualize antibody binding to polyGlcNAc-containing polymers, cells were reacted with the anti-human IgG antibodies conjugated to Alexa Fluor-488 (green fluorescence). DAPI was used to stain bacterial DNA (blue fluorescence). To visualize GlcNAc residues cells were also treated WGA conjugated to Texas Red (red fluorescence) Scale bar: 20 μm. (C) Colony immunoblot (top panel) of E. faecalis mutant and their parental strain grown on MOLP for 24 hours. S. aureus MN8 was used as positive control. The relative intensity obtained upon hybridization with mAb F598 was calculated for each colony using Image J (lower panel); (mean±SE; n = 8; ns, P>0.05 for both the overall one-way ANOVA and Tukey’s multiple comparison test). Scale bar: 1,000 μm. (D) Analysis of MOLP penetration (inside) by E. faecalis OG1RF WT and mutant ΔepaB. (PDF) [file ppat.1007571.s006.pdf]
