# Supplementary material for: PolyGlcNAc-containing exopolymers enable surface penetration by non-motile Enterococcus faecalis
Source: PLoS Pathog. 2019 Feb 11;15(2):e1007571. doi: 10.1371/journal.ppat.1007571 (PMC6386517; doi:10.1371/journal.ppat.1007571)
Supplement: S1 Table — Mean±SE; n = 6; *P<0.05; **P<0.01; ***P<0.001; **** P<0.0001 for both the one-way ANOVA and Tukey’s multiple comparison test; nonsignificant, ns. (PDF) [file ppat.1007571.s007.pdf]

S1 Table. One-way ANOVA and Turkey's multiple comparison test of the non-invading cells.

Tukey's multiple comparisons test	Summary	Adjusted P Value
WT vs. *glnA*::TnM	ns	>0.9999
WT vs. *rpiA::*TnM	ns	0.6291
WT vs. WT pAT28	ns	0.9077
WT vs. *glnA*::TnM pAT28	ns	>0.9999
WT vs. *rpiA*::TnM pAT28	ns	0.4035
WT vs. *rpiA*::TnM p-rpiA	ns	0.0606
WT vs. *glnA*::TnM p-glnA	ns	0.0779
glnA::TnM vs. *rpiA*::TnM	ns	0.8454
glnA::TnM vs. WT pAT28	ns	0.986
glnA::TnM vs. *glnA*::TnM pAT28	ns	0.9999
glnA::TnM vs. *rpiA*::TnM pAT28	ns	0.6426
glnA::TnM vs. *rpiA*::TnM p-*rpiA*	*	0.0236
glnA::TnM vs. *glnA*::TnM p-glnA	*	0.0311
rpiA::TnM vs. WT pAT28	ns	0.9994
rpiA::TnM vs. *glnA*::TnM pAT28	ns	0.6105
rpiA::TnM vs. *rpiA*::TnM pAT28	ns	>0.9999
rpiA::TnM vs. *rpiA*::TnM p-*rpiA*	***	0.0004
rpiA::TnM vs. *glnA*::TnM p-glnA	***	0.0005
WT pAT28 vs. *glnA*::TnM pAT28	ns	0.8974
WT pAT28 vs. *rpiA*::TnM pAT28	ns	0.9856
WT pAT28 vs. *rpiA*::TnM p-*rpiA*	**	0.002

WT pAT28 vs. *glnA*::TnM p-*glnA*	**	0.0027
glnA::TnM pAT28 vs. rpiA::TnM pAT28	ns	0.3866
glnA::TnM pAT28 vs. *rpiA*::TnM p-*rpiA*	ns	0.0648
glnA::TnM pAT28 vs. *glnA*::TnM p-glnA	ns	0.0832
rpiA::TnM pAT28 vs. *rpiA*::TnM p-rpiA	***	0.0001
rpiA::TnM pAT28 vs. *glnA::*TnM p-*glnA*	***	0.0002
rpiA::TnM p-*rpiA* vs. *glnA*::TnM p-glnA	ns	>0.9999

Mean±SE; n=6; *P<0.05; **P<0.01; ***P<0.001; **** P<0.0001 for both the one-way ANOVA and Tukey's multiple comparison test; nonsignificant, ns.
