# Supplementary material for: PolyGlcNAc-containing exopolymers enable surface penetration by non-motile Enterococcus faecalis
Source: PLoS Pathog. 2019 Feb 11;15(2):e1007571. doi: 10.1371/journal.ppat.1007571 (PMC6386517; doi:10.1371/journal.ppat.1007571)
Supplement: S2 Table — (PDF) [file ppat.1007571.s008.pdf]

S2 Table. List of putative genes evaluated by Nanostring analysis.

V583 ID#	MMH594 ID#	DESCRIPTION
EF0590	EOI84245.1	Polysaccharide deacetylase family
EF0887	EOI83927.1	Group 2 glycosyltransferase
EF2167	EOI81775.1	Group 2 glycosyltransferase
EF2176	EOI81783.1	Group 2 glycosyltransferase
EF2491	EOI79425.1	*cpsE*, group 2 glycosyltransferase
EF2492	EOI79426.1	*cpsD*, group 2 glycosyltransferase
EF2890	EOI79754.1	glycosyltransferase family
EF2891	EOI79755.1	glycosyltransferase family
EF2908	EOI79016.1	Group 2 glycosyltransferase
EF2198	EOI81802.1	*epaA*, group 4 glycosytransferase; Epa rhamnopolysaccharyde synthesis
EF2195	EOI81799.1	*epaD* in operon with *epaB and epaC*. Group 2 glycosyltransferases; Epa rhamnopolysaccharides synthesis
EF2190	EOI81802.1	*epaI*, group 2 glycosyltransferase
EF2180	EOI81787.1	*epaO*, in operon with 2181 (*epaN*), two group 2 glycosyltransferases. Epa rhamnopolysaccharides synthesis
EF2170	EOI81778.1	*epaX*, group 2 glycosyltransferase
EF3269	EOI78937.1	*folB*, folate biosynthesis; dihydroneopterin aldolase
EF0005	EOI85150.1	*gyrB*, DNA gyrase
EF1522	EOI81171.1	*sigA*, RNA polymerase sigma factorD
EF1192	EOI80855.1	*aqpZ*, aquaporin
EF3066	EOI78758.1	*def*, peptide deformylase
