# Supplementary material for: PolyGlcNAc-containing exopolymers enable surface penetration by non-motile Enterococcus faecalis
Source: PLoS Pathog. 2019 Feb 11;15(2):e1007571. doi: 10.1371/journal.ppat.1007571 (PMC6386517; doi:10.1371/journal.ppat.1007571)
Supplement: S3 Table — Ampr, ampicillin resistant, Rifr, rifampin resistant; Fusr, fusidic acid resistant; Spr, spectinomycin resistant; Tetr, tetracycline resistant; Cmr, chloramphenicol resistant; Emr, erythromycin resistant. E. faecalis MMH594 strains harboring pAT28 vector and derivatives required 750 μg/mL spectinomycin. Transposon insertion strains required 10 μg/mL chloramphenicol. Fluorescent reporter strains harboring pMV158 derivatives needed 15 μg/mL tetracycline. E. faecalis harboring pLT06 derivatives required 15 μg/mL chloramphenicol. pMINIMAD derivatives required 100 μg/mL ampicillin. WT: Wild type. (PDF) [file ppat.1007571.s009.pdf]

S3 Table. Strains and plasmid list used in this study.

Strain name	Parental background	Source	Description	Reference
Enterococcus faecalis				
DK11	JH2-2	Clinical	WT plasmidless strain derived from JH2	74
DK10	OG1RF	Oral	WT plasmid free, commensal; Rif^r, Fus^r	77
DK13	OG1RF	--	Δ*epaB*	Gilmore Lab
DK255	OG1RF	--	Δ*ebpABC*	26
DK256	OG1RF	--	Δ*ebpA*	26
DK29	OG1RF	--	pMV158-GFP, conjugation donor	78
DK30	V583	Blood	Clinical WT Van^r	75
DK1	VE14089	Blood	Plasmidless strain derived from V583	83
DK2	VE14089	--	Δ*epaX*	46
DK3	VE14089	--	Δ*epaX*::*epaX;* Erm^r	46
DK287	VE14089	--	WT, pMV158-GFP, Tet^r	This study
DK288	VE14089	--	Δ*epaX*, pMV158-GFP, Tet^r	This study
DK8	MMH594	Blood	Clinical WT Erm^r, Gen^r	76
DK250	MMH594	--	WT, empty pAT28 vector; Sp^r	This study
DK19	MMH594	--	WT, pMV158-GFP, Tet^r	This study
DK20	MMH594	--	WT, pMV158-mCherry, Tet^r	Gilmore lab
DK16	MMH594	--	*glnA*::TnM; Cm^r	This study
DK251	MMH594	--	*glnA*::TnM, empty pAT28 vector; Cm^r, Sp^r	This study
DK252	MMH594	--	*glnA*::TnM, pAT28-*glnRA*; Cm^r, Sp^r	This study
DK18	MMH594	--	*glnA*::TnM;pMV158-GFP; Cm^r, Tet^r	This study
DK14	MMH594	--	*rpiA*::TnM; Cm^r;	This study
DK253	MMH594	--	*rpiA*::TnM, empty pAT28 vector; Cm^r, Sp^r	This study
DK15	MMH594	--	*rpiA*::TnM, pAT28-*rpiA*; Cm^r, Sp^r	This study
DK17	MMH594	--	*rpiA*::TnM;pMV158-GFP; Cm^r, Tet^r	This study
DK267	MMH594	--	ΔEF2170	This study
DK21	1230	Clinical	WT	73
Escherichia coli				
DK91	DH5α	--	F$^-$ Φ80*lac*ZΔM15 Δ(*lacZYA-argF*) U169 *rec*A1 *end*A1 *hsdR*17 (rk$^-$, mk$^+$) *pho*A *sup*E44 λ$^-$*thi*$^-$1 *gyr*A96 relA1	Stratagene
Staphylococcus aureus				
DK43	MN8	Urogenital tract	WT	32
DK44	MN8	--	Δ*ica*, PNAG(PIA) deficient strain	32
Candida albicans				
DK80	SC5314	--	Prototrophic clinical isolate	80
**Plasmids**				
pMINIMAD			Thermosensitive plasmid; Amp^r	81
pLT06			Deletion construct used for amplification of Cm^r cassette (*camR*)	10
pIC333			Deletion construct used for amplification of Sp^r cassette (*spcR*)	79
pJR02			pMINIMAD vector with EF2170::*spcR* deletion construct; Amp^r	This study
pJR03			Derived from pJR02 with Cm^r from pLT06 vector; Amp^r, Cm^r	This study
pAT28			Complementation vector	82
pJR01			*glnRA* complementation vector	This study
pAH01			*rpiA* complementation vector	This study
pMV158			GFP cassette; Cm^r, Tet^r	92

Amp^r, ampicillin resistant, Rif^r, rifampin resistant; Fus^r, fusidic acid resistant; Sp^r, spectinomycin resistant; Tet^r, tetracycline resistant; Cm^r, chloramphenicol resistant; Em^r, erythromycin resistant. *E. faecalis* MMH594 strains harboring pAT28 vector and derivatives required 750 μg/mL spectinomycin. Transposon insertion strains required 10 μg/mL chloramphenicol. Fluorescent reporter strains harboring pMV158 derivatives needed 15 μg/mL tetracycline. *E. faecalis* harboring pLT06 derivatives required 15 μg/mL chloramphenicol. pMINIMAD derivatives required 100 μg/mL ampicillin. WT: Wild type.
