# Supplementary material for: PolyGlcNAc-containing exopolymers enable surface penetration by non-motile Enterococcus faecalis
Source: PLoS Pathog. 2019 Feb 11;15(2):e1007571. doi: 10.1371/journal.ppat.1007571 (PMC6386517; doi:10.1371/journal.ppat.1007571)
Supplement: S4 Table — Restriction sites are underlined (PDF) [file ppat.1007571.s010.pdf]

S4 Table. List of primers used in this study.

Name	Sequence 5' - 3'	Restriction Enzyme
JD1	CAGACGTTGAACGAGTTTTAG	
JD2	GGAGGATCCACCAGATTGGACGAAAGTAAC	BamHI
JD3	GCAATAACAGGTGCTTACTTTCATATCAATTTCCGCTACCTTC	
JD4	GAAGGTAGCGGAAATTGATATGAAAGTAAGCACCTGTTATTGC	
JD5	GGAAGACCAAACATAAGTAAAATCCACCTAGATCCTTTTGACTC	
JD6	GAGTCAAAAGGATCTAGGTGGATTTTACTTATGTTTGGTCTTCC	
JD7	GGAGGATCCCTTTTGGTACTTCAATATTCACTG	BamHI
JD8	CCTCAACATCTGGTCTCTTAG	
JD15	GAAGAATTCCAAATGATGGAATTACTCGAG	EcoRI
JD30	GGAGGATCCATTCATTCACAAACTAAAGAACG	BamHI
JD44	GTCGTCGACCAACGTGAATTTAGGTTTTGG	SalI
JD45	GTCGTCGACCGATTCACAAAAAATAGGCAC	SalI
HV172	CCAGGGATCCGTAAGTTGTGACCGATACCTG	BamHI
HV173	CCAGTCTAGACATTCTTAACGTGCGGGAAC	XbaI
TnMextF1	GAGAGCTTAGTACGTGAAACATG	
TnMextF2	AGCGACGCCATCTATGTGTCAGAC	

Restriction sites are underlined
